# Genetic Interaction Maps in *Escherichia coli* Reveal Functional Crosstalk among Cell Envelope Biogenesis Pathways

**DOI:** 10.1371/journal.pgen.1002377

**Published:** 2011-11-17

**Authors:** Mohan Babu, J. Javier Díaz-Mejía, James Vlasblom, Alla Gagarinova, Sadhna Phanse, Chris Graham, Fouad Yousif, Huiming Ding, Xuejian Xiong, Anaies Nazarians-Armavil, Md Alamgir, Mehrab Ali, Oxana Pogoutse, Asaf Pe'er, Roland Arnold, Magali Michaut, John Parkinson, Ashkan Golshani, Chris Whitfield, Shoshana J. Wodak, Gabriel Moreno-Hagelsieb, Jack F. Greenblatt, Andrew Emili

**Affiliations:** 1 Banting and Best Department of Medical Research, Donnelly Centre, University of Toronto, Toronto, Canada; 2 Department of Biology, Wilfrid Laurier University, Waterloo, Canada; 3 Hospital for Sick Children, Toronto, Canada; 4 Department of Biochemistry, University of Toronto, Toronto, Canada; 5 Department of Molecular Genetics, University of Toronto, Toronto, Canada; 6 Department of Biology and Ottawa Institute of Systems Biology, Carleton University, Ottawa, Canada; 7 Department of Microbiology and Molecular Genetics, Institute for Medical Research-Israel-Canada, The Hebrew University of Jerusalem, Jerusalem, Israel; 8 Department of Molecular and Cellular Biology, University of Guelph, Guelph, Canada; Agency for Science, Technology, and Research, Singapore

## Abstract

As the interface between a microbe and its environment, the bacterial cell envelope has broad biological and clinical significance. While numerous biosynthesis genes and pathways have been identified and studied in isolation, how these intersect functionally to ensure envelope integrity during adaptive responses to environmental challenge remains unclear. To this end, we performed high-density synthetic genetic screens to generate quantitative functional association maps encompassing virtually the entire cell envelope biosynthetic machinery of *Escherichia coli* under both auxotrophic (rich medium) and prototrophic (minimal medium) culture conditions. The differential patterns of genetic interactions detected among >235,000 digenic mutant combinations tested reveal unexpected condition-specific functional crosstalk and genetic backup mechanisms that ensure stress-resistant envelope assembly and maintenance. These networks also provide insights into the global systems connectivity and dynamic functional reorganization of a universal bacterial structure that is both broadly conserved among eubacteria (including pathogens) and an important target.

## Author Summary

Proper assembly of the cell envelope is essential for bacterial growth, environmental adaptation, and drug resistance. Yet, while the biological roles of the many genes and pathways involved in biosynthesis of the cell envelope have been studied extensively in isolation, how the myriad components intersect functionally to maintain envelope integrity under different growth conditions has not been explored systematically. Genome-scale genetic interaction screens have increasingly been performed to great impact in yeast; no analogous comprehensive studies have yet been reported for bacteria despite their prominence in human health and disease. We addressed this by using a synthetic genetic array technology to generate quantitative maps of genetic interactions encompassing virtually all the components of the cell envelope biosynthetic machinery of the classic model bacterium *E. coli* in two common laboratory growth conditions (rich and minimal medium). From the resulting networks of high-confidence genetic interactions, we identify condition-specific functional dependencies underlying envelope assembly and global remodeling of genetic backup mechanisms that ensure envelope integrity under environmental challenge.

## Introduction

The bacterial cell envelope serves as a resilient macrostructure and permeability barrier that protects microbes from osmotic stress, xenobiotics and environmental insults while supporting cell morphology and transport of essential nutrients and waste. For Gram-negative species like *Escherichia coli*, the cell envelope consists sequentially of a phospholipidic inner membrane (IM), a peptidoglycan (PG) cell wall embedded in aqueous periplasm, and an outer membrane (OM) composed of phospholipids and lipopolysaccharide (LPS). Unique sets of proteins determine the functional identity of each of these compartments under physiological demand [1], [2]. These include diverse outer membrane proteins (OMP) such as β-barrel porins that mediate cell adhesion and passage of small molecules, inner membrane proteins (IMP) involved in active transport and adaptation to changing growth conditions, and soluble periplasmic enzymes and membrane-tethered lipoproteins that process metabolic precursors required for envelope assembly. Proper expression, transport and activity of these envelope-associated proteins is critical to bacterial cell viability, morphology and stress-resistance [3].

The cell envelope also plays a crucial role in bacterial pathogenesis [3]. One-quarter of prescription antimicrobials are currently directed against proteins involved in envelope biogenesis [4], including classical antibiotics such as the β-lactams (e.g. penicillin) and modern ‘last resort’ glycopeptide drugs like vancomycin. Yet despite the emergence of widespread clinical resistance, few new therapeutics targeting the bacterial cell envelope have been developed over the past two decades due in part to the extraordinary functional robustness of this structure [5]. The utility of combination therapies targeting redundant pathways as an alternate clinical strategy [2] underscores the need to identify functional dependencies among essential envelope bioprocesses that contribute to envelope formation, stability and drug tolerance.

Cell envelope biogenesis has been studied extensively in *E. coli* [3], including investigations of individual genes and pathways involved in the biogenesis of fatty acids [6], cell wall [7], LPS [8] and OMP [9]. Yet important gaps still exist in the understanding of the global mechanisms that ensure faithful envelope assembly and integrity under different growth conditions or in response to antibiotic challenges [3]. New components of envelope assembly pathways continue to be discovered [10], [11], [12], [13], yet nearly one-third of the membrane proteins of *E. coli* are currently functionally unannotated likely in part due to incomplete or biased historical experimental analyses [1], [2], [14]. Moreover, most (>90%) of the annotated biosynthetic genes of *E. coli* are dispensable for viability under standard laboratory culture conditions [15]. This redundancy, which presumably reflects in part a robust modular systems level organization [16], has hindered both functional annotation [2] and antibiotic development using traditional single gene/target-centric approaches [17].

Unbiased genetic screens can reveal unexpected functional dependencies between genetic loci (i.e. epistasis, wherein the phenotypic effects of mutation of one gene are modified by one or more other genes). For example, aggravating (negative) genetic interactions, manifesting as synthetic sickness or lethality, often result from loss-of-function mutations in pairs of genes in parallel or compensatory pathways that impinge on a common essential process [18]. Conversely, alleviating (positive) interactions can occur between genes in the same pathway if the loss of one gene alone inactivates the pathway such that loss of a second gene confers no additional defect [18]. Large-scale genetic screens in yeast have outlined the tightly integrated functional organization of essential biological systems [19], [20], and global network rewiring in response to environmental stress causing DNA damage [21]. No analogous systematic surveys have yet been reported for bacteria, and only *ad hoc* genetic studies on a few select components of the *E. coli* cell envelope have been reported to date [22]. As a result, the degree of functional redundancy, connectivity and modularity among the biosynthetic pathways supporting envelope assembly and maintenance remains unclear. Such knowledge is paramount for targeting envelope systems resistant to existing antibiotics.

To this end, we applied our high-throughput synthetic genetic array (eSGA) screening technology [23] in a comprehensive manner to identify and quantify epistatic relationships between all known and predicted components of the cell envelope biosynthetic machinery of *E. coli* during growth in both auxotrophic and prototrophic culture conditions. Unbiased scoring and filtering of the resulting genetic data revealed condition-specific genetic interaction networks required for the proper formation and integrity of the OM, cell wall, and LPS, and functional dependencies mediating membrane protein secretion and cell division, which were verified independently. These functional association maps provide a unique perspective into the global functional architecture and dynamic rewiring of widely-conserved envelope bioprocesses critical to bacterial morphology, fitness and environmental adaptation. All of the data are publicly-accessible as a community resource *via* a dedicated web portal.

## Results

### High-throughput genetic screens of cell envelope bioprocesses

**Figure 1:**
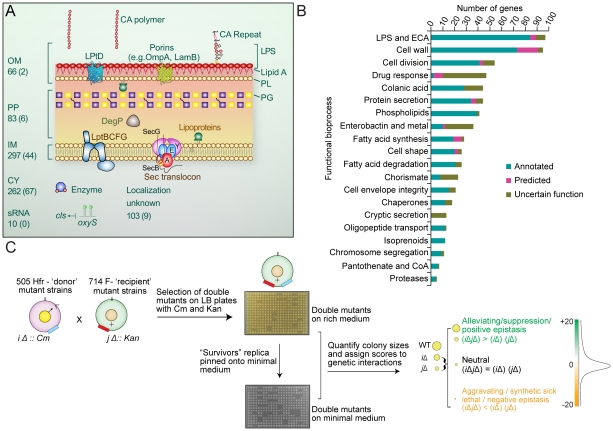
Cell envelope targets and schematic of cell envelope eSGA strategy. (A) Schematic summary of the eSGA gene targets associated with the *E. coli* outer membrane (OM), inner membrane (IM), periplasm (PP), cytoplasm (CY), regulatory RNA (sRNA), or of uncertain localization. Gene numbers are indicated, with essential genes shown in brackets. (B) Functional distribution of annotated (Group 1), uncertain function (Group 2) and predicted (Group 3) envelope target genes sorted into 20 broadly representative bioprocesses. (C) Schematic summarizing the experimental construction and computational analysis of two *E. coli* cell-envelope genetic interaction maps. After conjugation and genetic exchange, high density arrays of double-mutants were first selected on rich medium containing Cm and Kan. After outgrowth for 24 hours, surviving colonies were then replica pinned onto minimal M9 medium, to identify condition-dependent genetic interactions. Both sets of plates were digitally imaged and colony sizes quantified to determine gene pairs showing aggravating (synthetic lethal and synthetic sick) and alleviating (buffering) interactions.

Using a quantitative screening format originally developed to investigate pathway crosstalk in yeast [20], we performed 821 high-density eSGA screens to examine the fitness of all possible digenic mutant combinations of hypomorphic alleles (i.e. partial loss of gene function) of 128 essential biosynthetic genes and single-gene deletions of 683 non-essential protein-coding envelope genes and 10 small non-coding regulatory RNAs (sRNA) linked to post-transcriptional regulation of cell surface protein expression (Figure 1A, see Protocol S1). Target inclusion was based on an exhaustive survey of the literature and databases of envelope-related biosynthetic pathways and gene annotations [1] (Table S1).

Targets came from 20 representative bioprocesses (Figure 1B), including 363 integral membrane proteins (66 OMPs, 297 IMPs), and consisted of three basic types: Group 1 (Annotated), comprising 357 core biosynthetic genes (‘building block’ enzymes) with experimental evidence supporting direct involvement in a specific step of an annotated envelope biogenesis pathway or bioprocess; Group 2 (Uncertain Function), encompassing 286 genes, where existing experimental evidence indicates a role in envelope biogenesis but without certainty as to a particular pathway or bioprocess; Group 3 (Predicted), comprising 178 unannotated genes predicted to participate in envelope biogenesis based on protein-protein interactions, genomic context inferences [14], or tentative EcoCyc, Gene Ontology (GO) or GenProtEC assignments. Despite significant differences in bacterial morphology and envelope composition (e.g. Gram-positives versus Gram-negatives), many of these genes are broadly evolutionary conserved consistent with fundamental roles in cell envelope biology beyond *E. coli* (Figure S1 and Table S2).

Following the conjugation of single mutant strains and genetic transfer (Figure 1C), double mutants were first plated as replicate arrays onto solid agar containing rich (Luria Broth) medium. After outgrowth for 24 hrs at 32°C, viable stationary phase colonies were then replica pinned onto minimal (M9) medium to identify additional genetic interactions under limiting nitrogen and carbon levels (Figure 1C). Both sets of plates were digitally imaged, and colony sizes measured and normalized to account for experimental variation (see Protocol S2 and S3). Screen reproducibility was uniformly high (*r* typically >0.8; Figure S2A) and comparable to high-quality quantitative genetic interaction screens reported previously for yeast [19], [20].

Although the Hfr donor strain is not isogenic with the BW25113 KEIO deletion mutant strain background, a pilot test set of 30×30 conjugations showed that the growth rates of double mutants produced by crossing 30 diverse F- ‘recipient’ strains from the Keio collection [15] with the corresponding set of 30 ‘donor’ mutants in either an Hfr C or an isogenic strain background were comparable (*r* = 0.7; Figure S2B).

Digenic mutant fitness was estimated using an established multiplicative model [19] which reports both the strength and confidence of genetic interactions between any two genes (see Protocol S4). Briefly, if two genes are functionally unrelated, the growth rates of the respective single mutations are predicted to combine in a simple multiplicative manner in the double mutant; significant deviations from this expected fitness imply a functional association [18].

The resulting *E*-scores of the double mutants on both rich medium and minimal medium (Table S3) showed a bimodal distribution (Figure S2C), with the major peak approximating a normal distribution centered on neutrality, confirming the expectation that genetic interactions are relatively uncommon (i.e. limited to only certain gene pairs). Positive *E*-scores suggestive of alleviating interactions (double mutants grew more rapidly than expected) were found in the right tail of the distributions, reflecting factors operating in the same pathway [24], while aggravating interactions (double mutants grew more slowly than expected) occurred in the heavy left tail, with a prominent peak of highly negative *E*-scores representing gene pairs exhibiting synthetic lethality [19].

### Deriving high-confidence genetic interaction maps

Genetic interactions by a given biogenesis gene reflect its functional associations with other envelope components and thus serve as a high-resolution phenotype. We applied a statistical framework to define suitable *E*-score cutoffs to derive biologically-relevant interactions by minimizing the false discovery rate. Specifically, we evaluated the extent to which pairs of envelope genes involved in different biogenesis bioprocesses showed significant (*p*-value≤0.05) enrichment at various *E*-score values.

Enrichment increased progressively as the *E*-score threshold was raised (Figure S2D), reaching a maximum at −2 (aggravating interactions) and +2 (alleviating interactions). We used these apex values (*E*≤−2, *E*≥2) as cutoffs, and assigned *p*-values using a null distribution background (see Methods).

**Figure 2:**
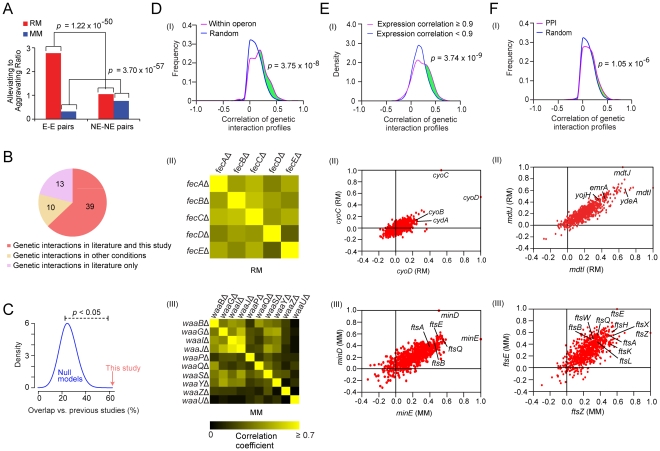
Analysis on the genetic interaction networks derived from two growth conditions. (A) Comparison of ratios of alleviating (*E-*score ≥2) to aggravating (*E-* score ≤−2) genetic interaction involving essential (E) and non-essential (NE) gene pairs on both growth conditions, *p*-values were computed using Fisher's exact test. (B) Venn diagram showing the overlap of the genetic interaction pairs derived from this study and the literature. (C) Significance testing of the agreement between the literature and this study (red arrow) compared against expected gene pair frequencies by randomly sampling (blue distribution represents 10,000 null models). (D) Distribution of correlations coefficients between the genetic interaction profiles for gene pairs within the same operon versus randomly drawn gene pairs (panel I). Pair-wise profile correlation coefficient of genes in the Fe-enterobactin “*fec*” operon in rich medium (panel II) and for LPS 1, 2-glucosyltransferase components of *waa* operon in minimal medium (panel III). (E) Distribution of genetic interaction profile correlation for gene pairs with correlated co-expression (panel I). Scatter-plot of correlated genetic profiles is shown for *cyoD* (*x*-axis) and *cyoC* (*y*-axis) in panel II, and for *minE* (*x*-axis) and *minD* (*y*-axis) in panel III. (F) Distribution of correlation coefficients between the genetic interaction profiles of gene pairs encoding interacting proteins versus randomly drawn gene pairs (panel I), where the *p*-value was computed using the two-sample Kolmogorov-Smirnov (KS) test. Scatter plot of correlated genetic profiles for *mdtI* (*x*-axis) and *mdtJ* (*y*-axis) (panel II). Scatter plot of correlated genetic profiles for *ftsZ* (*x*-axis) and *ftsE* (*y*-axis) (panel III).

Strikingly, the patterns of genetic interactions found in rich and minimal (Table S3) medium were markedly different, reflecting a profound reorganization in the envelope biogenesis machinery. For example, although more highly connected on average than non-essential genes (Figure S2E and S2F), essential genes exhibited a higher ratio of alleviating interactions (*p*-value = 1.22×10^−50^) in rich medium (Figure 2A, Figure S3A, Protocol S5). Conversely, aggravating interactions were far more common (*p*-value = 3.70×10^−57^) for essential genes in minimal medium (Figure 2A), suggesting compensatory relationships emerge under environmental constraint (i.e. nutrient limitation). The likelihood of a genetic interaction by any one particular envelope biogenesis gene in either condition was correlated both with gene essentiality and mRNA expression levels in culture (Figure S3B). As with yeast [19], [25], essential genes producing abundant transcripts had significantly more aggravating interactions, while envelope factors with multiple annotated functions suggestive of pleiotropy were more likely to display synthetic lethality (Table S4).

To independently gauge the accuracy of these networks, we examined the screen results obtained for 62 gene pairs reported previously to exhibit aggravating (53 pairs) or alleviating (9 pairs) phenotypes. In total, we correctly captured two-thirds (63%) of the published GI (Figure 2B and Table S5), which is statistically significant (*p*-value<0.05 by random sampling; Figure 2C). For example, the rich medium network recapitulated aggravating interactions between the periplasmic chaperone *surA* and the β-barrel protein assembly machinery (BAM) [26] and a functionally redundant chaperone *skp* [27]. Conversely, the minimal medium network captured the conditional synthetic lethality reported between cell division proteins *zapB* and *ftsZ* [28]. Half (10 of 23) of the discordant gene pairs were found using different growth conditions in the literature (Table S5), reinforcing the notion of conditional-dependency.

Many components of the cell envelope biosynthetic machinery are predicted to be functionally-associated based on physical association (Figure S4A), transcriptional co-expression (Figure S4B), and/or genomic context (Figure S4C). Consistent with this, cell envelope genes with positively correlated genetic profiles were more likely to be present within the same operon in *E. coli* compared to random pairs of genes (Figure 2D), consistent with the natural chromosomal clustering of functionally-related genes in bacteria [29]. For example, components of the *fec* Fe-enterobactin uptake system were correlated in rich medium (Figure 2D), consistent with joint participation in the import of cellular iron [30]. Similarly, the genetic interaction patterns of the LPS 1, 2-glucosyltransferase components of *waa* operon (formerly *rfa*) were correlated in minimal medium (Figure 2D), consistent with the coordinated role in LPS core biosynthesis [31]. Likewise, pairs of co-expressed envelope genes also tended to show more positively correlated genetic interaction profiles (Figure 2E). For example, the genetic interaction profiles of the co-expressed cytochrome *bo* terminal oxidase subunits (*cyoC*, *cyoD*) were correlated in rich medium (*r* = 0.53; Figure 2E), reflecting elevated aerobic respiration. In contrast, the genetic interaction patterns of the co-expressed cell division components (*minD*, *minE*) were closely correlated in minimal medium (*r* = 0.51; Figure 2E), consistent with the cooperative role in modulating the division potential of cellular sites located at mid cell and at the cell poles [32].

As in yeast [19], [20], envelope biogenesis factors with highly correlated genetic profiles were also significantly more likely to be connected by protein-protein interaction (PPI) (Figure 2F). For example, the genetic profiles of two transporters *(mdtI, mdtJ)* that form a heterodimeric complex required for spermidine excretion [33] were highly correlated in rich medium (*r* = 0.65; Figure 2F). The profiles of *mdtJ*/*mdtI* were also similar to other translocases linked to multidrug resistance such as resistance-nodulation-cell division, major facilitator, and ATP-binding cassette proteins (Figure S5). Conversely, the genetic profiles of *ftsE* and its interacting partner *ftsZ*, which are required for assembly of the cytokinetic Z-ring [34], were closely correlated in minimal medium (*r* = 0.60; Figure 2F).

We concluded that the filtered networks reliably captured functional dependencies, and hence could be used to infer biologically-relevant relationships. For example, the genetic profile of the recently characterized OM lipoprotein *ycfM*/*lpoB* was similar to that of the penicillin binding protein *mrcA* under both rich and minimal conditions (Table S6), consistent with a recently proposed role as joint regulators of PG synthesis [12].

### Bioprocess coupling

**Figure 3:**
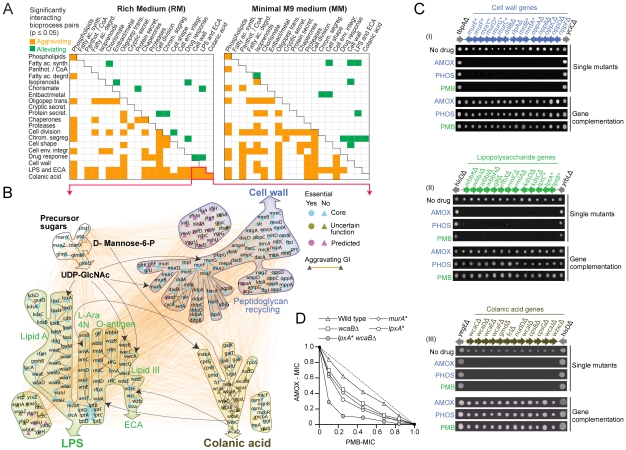
Bioprocesses crosstalk critical for envelope integrity. (A) Distinct patterns of enrichment in aggravating and alleviating genetic interactions between select envelope bioprocesses in rich and minimal medium. (B) Aggravating genetic interactions (orange edges) and metabolic links (black arrows) between the CA, LPS/ECA and cell wall biosynthetic pathways. (C) Antibiotic sensitivity of select *E. coli* mutants; bioprocess and gene identity indicated. Phenotypic complementation based on plasmid rescue. (D) Isobologram portraying the enhanced combination potency of amoxicillin (AMOX) and phosphomycin (PMB) against *E. coli* mutants deficient in cell wall (*murA*), LPS (*lpxA*) or CA (*wcaB*) production. Dotted line indicates additivity; MIC, minimal inhibitory concentration normalized relative to no drug control.

Having established that the genetic maps were informative about biological relationships at the level of individual envelope components, multiprotein complexes and biosynthetic pathways, we explored the global functional connections linking envelope bioprocesses in the two networks (Table S7 and S8). Using functional enrichment analysis (see Materials and Methods), we identified significant (*p*-value≤0.05) crosstalk (elevated patterns of aggravating or alleviating interactions) between bioprocesses (Figure 3A).

We found that certain patterns were prevalent in both culture conditions (Figure 3A). For example, aggravating interactions were prominent (*p*-value≤0.05) between the oligopeptide transport, cell wall, cell shape and cell division machineries, consistent with the tight integration of these bioprocesses. As bacteria proliferate, the murein sacculus elongates in such a way that the distinctive rod shape of *E. coli* is maintained [7]. Murein peptides liberated by dynamic degradative recycling of the cell wall are actively imported for reuse by multiple reuptake systems [7]. Defective oligopeptide transport exacerbates slow growth and morphological deficiencies [7], which is reflected in our genetic networks.

In some cases, this crosstalk was unexpected. For example, Figure 3B shows a sub-network of aggravating interactions between the pathways generating colanic acid (CA), an exopolysaccharide (also known as M-antigen) produced by *E. coli* K-12 in response to hypotonic conditions or membrane perturbations [35], and the pathways responsible for producing the cell wall, LPS and enterobacterial common antigen (ECA), another surface glycophospholipid [36]. This crosstalk presumably reflects use of shared metabolic intermediates. For example, UDP-N acetylglucosamine is required for biosynthesis of ECA, LPS (lipid A) and PG in *E. coli* [36]. While disturbances in envelope integrity can lead to increased CA synthesis *via* activation of the Rcs regulon [37], defects in LPS formation do not affect CA expression under standard laboratory growth conditions [38], and dependencies between these systems have not been reported before [37].

To verify our results, we challenged CA mutants with amoxicillin and phosphomycin, antibiotics, which block cell wall/PG biogenesis, or with polymyxin B, which targets the lipid A component of LPS [2]. Consistent with our genetic data, CA strains were nearly as hypersensitive to the compounds as mutants deficient in cell wall and LPS formation (Figure 3C). A synergistic inhibitory effect was also observed when CA/LPS double (*wcaB lpxA*) mutants were challenged with amoxicillin and polymyxin B simultaneously (Figure 3D). Collectively, these data indicate that an underlying functional coordination (direct or indirect) of envelope glycoconjugate production is essential to sustain envelope integrity.

### Conditional rewiring of cell envelope bioprocesses

Comparison of the two networks revealed differential physiological demands placed by the two culture conditions. For instance, alleviating interactions were preferentially detected in rich medium (*p*-value = 1.42×10^−3^; Figure 3A) between the pathways producing fatty acids and chorismate, the last branch point of the shikimate pathway that produces aromatic amino acid precursors. When amino acid pools exceed the requirements for protein synthesis, surplus aromatic precursors are directed towards fatty acid biosynthesis [39]. Hence, the lipid composition of the bacterial cell envelope reflects the combined output of both the shikimate and fatty acid pathways, which is mirrored as an alleviating phenotype during double mutant growth in abundant nutrient conditions.

**Figure 4:**
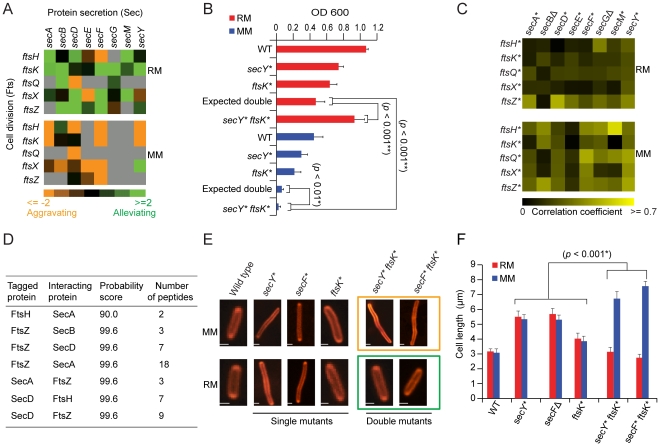
Genetic dependencies reflect functional relationships. (A) Genetic interaction patterns between secretory and cell division factors. (B) Growth rates (fitness) of *secY* and *ftsK* single and double mutants in liquid rich (RM) or minimal medium (MM) at 32°C over 24 h; hypomorphic alleles indicated by asterisks. (C) Pair-wise correlation coefficient profiles of secretory and cell division mutants grown on rich medium or minimal medium. (D) Identification of Sec proteins co-purified from the endogenous affinity tagged Fts protein by tandem mass spectrometry. (E) Fluorescent microscopy of *E. coli* stains (stained with FM4-64) after growth in minimal or rich medium; scale-bars 2 µm. (F) Quantitative measurements of changes in mutant cell length after growth in rich and minimal medium. Error bars show measurement of standard deviation from three independent experiments.

Conversely, alternate selective pressures were placed on envelope biosynthetic pathways in minimal medium, where aggravating interactions became more pronounced (*p*-value = 9.4×10^−4^) between the cell division (Fts) and protein secretion (Sec) systems (Figure 4A). For example, SecY and Ftsk mutants showed alleviating interactions on rich medium, but synthetic lethality on minimal medium. This differential dependency was confirmed by liquid culture growth curve assays (Figure 4B). The genetic interaction profiles of cell division and secretion mutants were also more highly correlated on minimal medium than rich medium (Figure 4C). Genes exhibiting alleviating interactions are more likely to encode proteins that are physically associated [40]. Consistent with this, we found that affinity purified endogenous Fts interacts physically with the Sec export apparatus (Figure 4D, see Methods), implicating the Sec translocon directly in targeting of critical cell division determinants.

Protein secretion is an important determinant of nutrient import, and is known to be slowed along with cell division when bacteria are grown with limiting nutrients, favoring cell elongation [41]. Moreover, the cell surface localization of several Fts septation proteins including the essential *ftsI* protein (penicillin-binding protein 3) with a membrane spanning segment is one such process that requires *sec* translocon gene functions for correct membrane integration [41], [42]. Consistent with this, cell division/secretion double mutants formed exaggerated filamentous cell aggregates in minimal medium, whereas this phenotype was rescued by growth in rich medium (Figure 4E and 4F), suggesting transport of essential cleavage factors is limiting in nutrient-poor conditions.

Using a variant of differential interaction network strategy [21], slightly over 1, 400 gene pairs showed contrasting genetic interactions in rich versus minimal medium (Table S9, Protocol S6). These differential interactions were enriched significantly (*p*-value≤0.05) for genes functioning in cell division, chromosome segregation and protein secretion pathways (Figure S6A). For example, annotated murein amidases (e.g. *amiABC*) involved in septum splitting, and cell division proteins localized to the membrane-associated ring structure (e.g. *ftsAEKHLQXYZ*) showed differential interactions (i.e. aggravating in minimal and alleviating in rich medium) (Figure S6B). Conversely, we observed significantly fewer differential interactions (*p*-value≤0.05) among genes annotated to fatty acid degradation, stress response and chaperone pathways (Figure S6A), suggesting that functional connections between these genes remains to a larger extent unchanged.

### Functional coupling of murein hydrolases to cell septation

**Figure 5:**
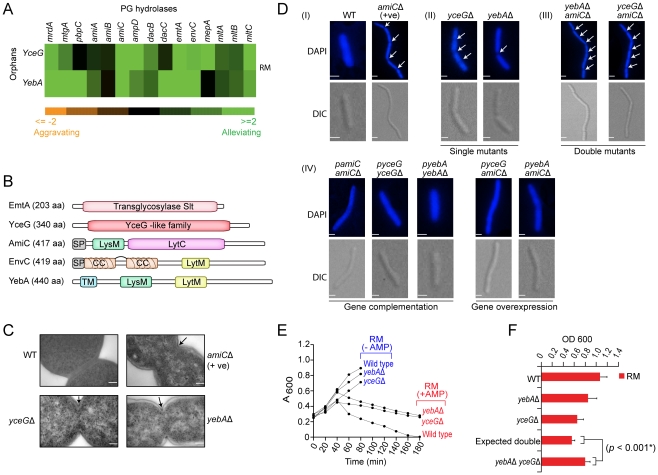
Novel hydrolase factors in septal PG splitting. (A) Alleviating genetic interactions between annotated PG hydrolases and YceG/YebA. (B) Protein architecture (amino acid length indicated); domain structure of YceG unknown. CC, coiled coil; TM, transmembrane helix; SP, signal peptide. (C) Electron microscopy showing impaired membrane invagination and uncleaved PG septa (arrows) in *E. coli* mutants relative to wild-type cells; scale-bar 500 nm. (D) Cell division defects (arrows), phenotypic complementation (plasmid-based wild-type gene copy) or transgenic rescue. Strains stained with DAPI were visualized using a high content microscopy with differential interference contrast (DIC) and fluorescence optics; scale-bar 2 µm. (E) Differential strain growth with or without ampicillin (AMP, 5 µg/ml). (F) Alleviating double mutant interaction in liquid rich medium at 32°C over 24 h.

Since functionally-related genes often have similar genetic profiles [19], [20], [40], the biological roles of incompletely characterized components can potentially be inferred based on correlation to annotated components. An illustrative example is a sub-network of strong alleviating interactions found in rich medium between the unannotated genes *yceG* and *yebA* and with several well-known cell division PG hydrolases (Figure 5A), each with distinct domain-architectures (Figure 5B). Binary fission in *E. coli* and other Gram-negatives depends on localized cell wall assembly at the division site by PG synthases e.g., penicillin-binding proteins [43]; subsequent splitting of these septa by PG hydrolases is required for cell division [44], [45]. Consistent with our genetic data, ultrastructure analysis revealed impaired membrane invagination and cytokinesis in *yebA* and *yceG* mutants (Figure 5C), with chains of daughter cells connected by uncleaved septa (Figure 5D and Figure S7A). Over-expression of either factor *in trans* fully rescued the phenotype (Figure 5D). Single *yebA* and *yceG* mutants also showed reduced sensitivity to ampicillin (Figure 5E), which blocks PG assembly, whereas wild-type *E. coli* rapidly lyse due to imbalanced PG hydrolase activity [44]. Consistent with joint promotion of septal PG splitting, growth curve assays confirmed an alleviating interaction between *yebA* and *yceG* in rich medium (Figure 5F).

### Novel factors for OM integrity

**Figure 6:**
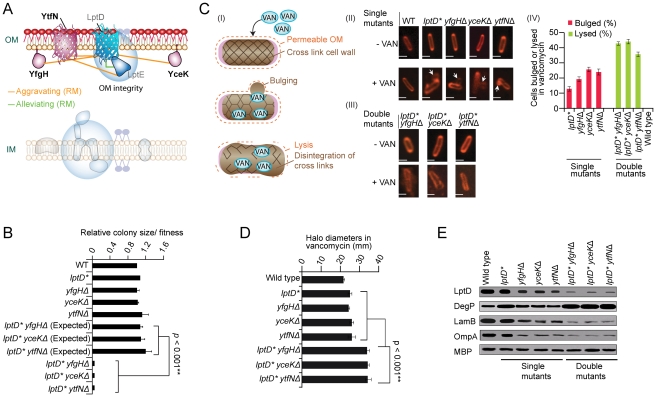
Novel factors required for OM integrity. (A) Genetic interaction sub-network in rich medium. (B) Aggravating interactions between *lptD* and *yfgH*, *yceK*, and *ytfN*. (C) Proposed mechanism of drug hypersensitivity (I) modeled on [68]: envelope deficiency results in a porous OM (dashes), thereby allowing vancomycin (VAN) into the periplasm where it blocks PG cross-linking, weakening the cell wall and resulting in cytoplasmic membrane bulges in single mutant strains (II) or catastrophic cell wall failure and lysis in double mutants (III); scale bar equals 2 µm. Quantification (percentage) of bulged or lysed cells following vancomycin treatment according to strain genotype; error bars indicate standard deviation (IV). (D) Growth inhibition of strains treated with vancomycin. Graph shows mean drug disk halo diameter and standard deviation (error bars) in three independent experiments; *p*-value calculated using Student's *t*-test. (E) SDS-PAGE immunoblot analyses of marker protein levels in indicated strains during logarithmic growth in rich medium; periplasmic maltose-binding protein (MBP) used as loading control.

A sub-network of strong aggravating interactions was also found in rich medium (Figure 6A and 6B), linking 3 unannotated genes (one encoding a putative β-barrel protein, *ytfN* and the other two lipoproteins, *yfgH*, *yceK*) to the *lptDE* complex involved in the final stages of LPS assembly at the OM outer leaflet [46]. Since impaired LPS destabilizes the envelope integrity [10], we challenged the mutant strains with vancomycin, an inhibitor of PG formation normally excluded by the intact OM of Gram-negatives [47]. Unlike drug-resistant wild-type *E. coli*, vancomycin induced morphological defects manifested by prominent mid-cell membranous bulges in single mutants and cell wall failure/lysis in double mutants (Figure 6C). Impaired OM integrity was also evident by drug hypersensitivity (Figure 6D), lowered OMP abundance (Figure 6E), and elevated levels of the σ^E^-stress response DegP protease [10]. Taken together, these data point to the tight participation of *yfgH*, *yceK*, *ytfN* in LPS/OM formation and OM integrity

### YhjD participates in LPS transport

**Figure 7:**
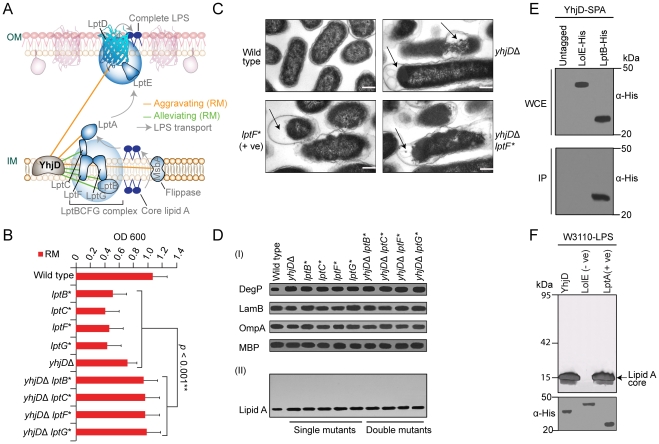
YhjD is required for LPS transport. (A) Genetic interaction sub-network (rich medium) involving LPS transport factors. LPS is synthesized at the IM cytoplasmic face, flipped across the bilayer by MsbA, extracted by LptBCFG, and transported to the OM by LptA for final assembly by LptDE. (B) Strain growth in rich liquid medium at 32°C over 24 h. (C) Electron microscopy showing morphological alterations (arrows) in mutant *E. coli* strains relative to wild-type cells; scale-bar 500 nm. (D) SDS-PAGE immunoblots of marker proteins (I) and silver staining LPS (II). (E) Co-immunoprecipitation of SPA-tagged YhjD with His_6_-tagged LPS transport component. (F) LPS-binding by immobilized recombinant YhjD (test) and LptA (positive control) but not LolE (negative control). After extensive washing, bound LPS molecules were visualized by SDS-PAGE and silver staining.

Another envelope gene of uncertain function, *yhjD*, which encodes a putative IMP with (5–6 TMH) previously implicated in Lipid A precursor IV_A_ transport [48], showed a similar genetic profile (Table S6) and alleviating interactions (Figure 7A and 7B) with components of the *lptBCFG* complex that extracts LPS intermediates from the IM for passage to the OM [49], and aggravating interactions with other LPS transport factors (Figure S7B and S7C).

Mutant *lptBCFG* strains have morphologically perturbed envelopes [10], [50]. Transmission electron microscopy (TEM) likewise revealed a defective ultrastructure, membranous projections and periplasmic bodies characteristic of compromised LPS transport in strains lacking *yhjD* (Figure 7C). Like *lptBCFG* mutants [10], [50], strains lacking *yhjD* also showed an activated DegP stress response and accumulated intracellular lipid A precursors whereas OMP abundance was not affected (Figure 7D). Consistent with the alleviating epistasis, these defects were not exacerbated in *yhjD lptBCFG* double mutants.

Since alleviating interactions arise with in pathways or physical complexes [40], we affinity purified endogenous YhjD from detergent solubilized *E. coli* cell extracts to identify stably associated proteins (see Methods). As shown in Figure 7E, YhjD co-purified specifically with LptB, consistent with association as a multiprotein complex. Moreover, recombinant YhjD also bound selectively to core-lipid A (rough LPS) *in vitro* (Figure 7F). Since site-specific suppressor mutation in YhjD has been shown to reduce the toxic-side effects of lipid IV_A_ accumulation by rendering the LPS transporter MsbA critical for lipid IV_A_ trafficking [48], it is conceivable that the deletion of YhjD eliminates the buildup of toxic lipid A intermediates in *lptBCFG* mutants, resulting in alleviating phenotype.

### Genetic networks capture regulatory relationships

Antisense sRNAs regulate gene expression by base-pairing to mRNAs *via* imperfect complementarity [51], often in response to environmental contingency such as stationary phase cells in nutrient rich medium or in minimal medium challenge [52], [53]. We therefore examined the genetic interactions of the 10 sRNAs in our networks (*cyaR*, *dicF*, *gcvB*, *micA*, *micC*, *micF*, *omrA*, *oxyS*, *rybB*, *sgrS*) with both verified and computationally-predicted targets (Table S10).

**Figure 8:**
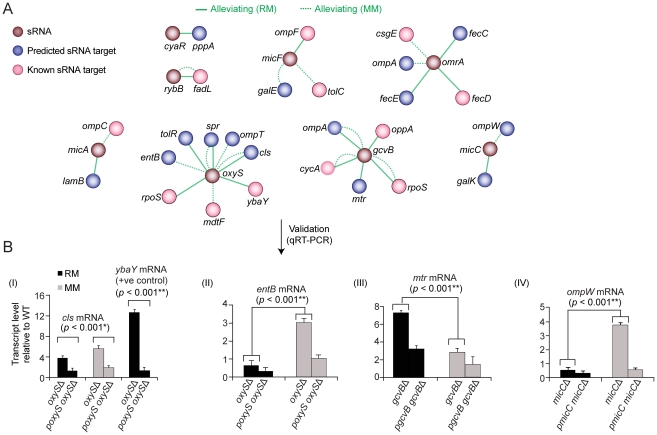
Target-regulator genetic interactions. (A) Alleviating interactions between known (pink) or predicted (blue) gene targets and cognate sRNA regulators (brown). (B) Quantitative real-time PCR analysis of target transcript levels in regulator mutants on rich or minimal medium. Values indicate mean fold-change ± standard deviation (error bars) of triplicate biological measurements normalized to a housekeeping gene product (glutathione *S*-transferase).

Cognate target-regulator pairs showed predominantly alleviating phenotypes under both culture conditions (Figure 8A). For example, *oxyS* with both the OM lipoprotein *ybaY* [54] and *cls*, which encodes cardiolipin synthase, whose levels are both significantly elevated in *E. coli* mutants lacking *oxyS* in rich and minimal medium (Figure 8B). However, condition-specific regulator-target relationships were also observed consistent with differential sRNA activity (Figure 8B). For instance, alleviating interactions were preferentially observed in rich medium between *gcvB* and *mtr* stationary phase culture cells, which encodes a tryptophan-specific permease feedback inhibited *via* the Trp repressor [55], and *oppA*, which encodes a periplasmic oligopeptide transporter. Deletion of *gcvB* causes constitutive expression of *oppA* [56], which is normally repressed in nutrient-rich conditions. Similarly, *omrA* displayed an alleviating phenotype in rich medium with iron transporters (*fecCDE*). Although essential for growth, iron is toxic and its import is tightly control [57].

Conversely, the regulators *micC* and *oxyS* displayed alleviating interactions with certain targets preferentially in minimal medium (Figure 8A), implying repression is lost in nutrient-limiting conditions. We confirmed that the mRNA levels of *ompW* and *entB* increase substantively in minimal medium in *micC* and *oxyS*, mutants, respectively (Figure 8B). Collectively, these results demonstrate how quantitative genetic interaction maps can be used to probe regulatory relationships orchestrating cell envelope homeostasis.

## Discussion

The cell envelope forms an essential interface between a microbe and its environment, or host in the case of pathogens. While *E. coli* has clearly evolved well-developed adaptive mechanisms to withstand diverse perturbations [2], our genetic results show that the K-12 laboratory isolate is unable to compensate for the loss of different combinations of individual cell envelope components and bioprocesses in culture. We have provided diverse lines of evidence supporting the unexpected biological participation of multiple novel factors in disparate core envelope processes. While additional follow-up mechanistic studies are warranted, the genetic interaction networks serve as a valuable resource for gleaning mechanistic and cell biological insights into *E. coli* envelope bioprocesses at the gene, pathway and physiological level.

These genetic interactions networks also inform on the global modular biological architecture of the bacterial cell envelope biosynthetic machinery, and its pronounced re-organization as an adaptive environmental response. Although our screens missed additional cases of epistasis emergent under alternate growth or stress conditions, the size and scope of the genetic map provides a fresh perspective into the functional interplay between convergent and compensatory systems that collectively ensure cell envelope assembly and integrity under two commonly studied culture conditions. The functional relationships we detected extend beyond established biochemical pathways and metabolic fluxes, and are consistent with the recently introduced notions of conditional and induced essentiality [58] and the substantive rewiring of genetic interaction networks in yeast following genotoxic stress [21]. Although the evolutionary basis for these emergent systems properties is unclear, it suggests a ubiquity of dynamic bioprocess crosstalk among microbes [3].

*E. coli* has an envelope architecture that is very similar to that of infectious species like *Salmonella enterica* (serovar *Typhimurium*), *Pseudomonas aeruginosa* and *Neisseria meningitidis.* Just as genetic screens in model eukaryotes have facilitated the characterization of conserved disease-related biological systems in humans [18], the generation of genetic interaction maps for *E. coli* suggests functional vulnerabilities that could potentially be exploited to combat these and other pathogens. For example, the coupling of cell wall and LPS glycoconjugate production we observed implies that a new family of peptidomimetic antibiotics (e.g. protegrin) targeting LPS assembly in *P. aeruginosa* [59] might be potentiated by inhibitors of either PG assembly or exopolysaccharide virulence factors analogous to CA in *E. coli*.

To facilitate exploration of these connectivity maps, our datasets are publicly accessible *via* a dedicated web portal (http://ecoli.med.utoronto.ca/eMap/CE/). The fact that a wide breadth of biological information could be derived from a defined collection of double mutants using a simple measure of colony growth provides strong motivation for future efforts aimed at examining other bacterial traits of broad biological, clinical and pharmacological significance.

## Materials and Methods

### Functional annotations

*E. coli* cell envelope gene targets suitable for screening were compiled based on published functional studies and database surveys [1], pathway annotations in EcoCyc, and relevant GO and GenProtEC annotations. Literature curated genetic interactions were manually compiled from low-throughput experimental studies. To generate functional association networks (see Protocol S7 and S8), high-quality *E. coli* PPI curated in the eNET, BIND, DIP, IntAct and MPI_LIT databases were compiled; microarray based mRNA transcript profiles were downloaded from the M3D database to derive a network of co-expressed genes based on correlation; and functional connections inferred by genomic context methods were computed as reported before [14]. Metabolic networks were reconstructed using Ecocyc annotations.

### Strains, plasmids, and genetic screens

Bacterial strains and plasmids used in this study are listed in Table S11. The F- ‘recipient’ single gene deletion mutants were from the Keio knock-out strain collection [15], marked with kanamycin resistance (Kan^R^), and from the recently developed ASKA single-gene deletion mutant library (Yamamoto et al., unpublished data) marked with chloramphenicol-resistance (Cm^R^). Donor mutant strain construction, conjugation and imaging was performed essentially as described [23]. Briefly, to construct Hfr Cavalli (Hfr C) donor knockout mutants, λ-Red recombination was used to replace the entire coding sequence of each open reading frame of non-essential genes with a drug selection marker, whereas the selection cassette was integrated into 3′-UTR of essential genes to perturb transcript abundance [23]. In total, 505 ‘query’ gene mutations in Hfr C were systematically transferred *via* conjugation to an arrayed collection of 714 F- ‘recipient’ knockout and hypomorphic mutant strains [23]. Robotic colony pinning and drug selection procedures are detailed in Protocol S2. An “Hfr” donor (marked with Kan^R^) isogenic with the *E. coli* single-gene deletion knock libraries was constructed for testing essentially as described [60].

### Bioinformatics analysis

Plate image processing and colony size quantization were adapted from an automated image processing system originally devised for yeast [61]. Epistasis scores were calculated based on a multiplicative model [61] where a GI between a pair of genes (*i*, *j*) is identified if the fitness phenotype of the double mutant (*Wij*) deviates significantly from that predicted for non-interacting gene pairs (*Wi*×*Wj*). Enrichment was determined by contrasting the frequency of observed genetic interactions between or within functional modules against the expected frequencies according to the hypergeometric distribution using the Fisher's exact test [62] with correction for multiple hypothesis testing [63]. This enrichment approach was used to determine the optimal *E*-score cutoff values for selecting aggravating and alleviating genetic interactions. Additional details are provided in Protocols S2, S3, S4, S5, S6, S7.

Computational prediction of regulatory sRNA targets was performed using RNAup [64] from the Vienna RNA package (-Xp-w20-b parameters) to measure sequence complementarity between each sncRNA and the 5′ regions (−50 and +50 nt relative to translation start site) of all protein coding genes in *E. coli*. Genes were ranked according to free energy, with the lowest scoring predicted as targets. Known mRNA targets were compiled from published functional studies and the NPInter and sRNAMap databases.

### Western blotting and protein affinity purification

Immunoblots were probed with polyclonal (LptD, OmpA, LamB, DegP) or monoclonal (MBP, LPS, His epitopes) antisera. Hexahistidine (His_6_)-tagged *E. coli* LptB or LolE fusion protein were expressed using an IPTG inducible promoter from a high copy pCA24N plasmid [65]. Sequential peptide affinity (SPA) tagging and purification of Fts, Sec and YhjD was performed essentially as previously described [66]. The affinity-purified proteins were subjected to gel-free liquid chromatography-electrospray-linear ion trap tandem mass spectrometry (LC-MS) to identify the stably and weakly associated interacting proteins with high sensitivity. Spectral searches were performed against a database containing complete set of *E. coli* protein-coding sequences and filtered for high-confidence matches essentially as described [66]. Details of the immunoprecipitation are provided in Protocol S9.

### Phenotypic and drug interaction assays

For growth curve analyses, overnight cultures were inoculated into 100-well microtitre plates containing 100 µl of liquid medium, incubated with shaking at 32°C for over 24 hrs with optical density (600 nm) measured every 15 min using an automated Bioscreen-C (Thermolabsystems, Helsinki, Finland). For drug assays, overnight *E. coli* cultures in liquid LB medium were serially diluted and pinned onto solid LB agar plates in the absence or presence of drug (see Protocol S10). To examine cell morphology, strains were grown to log-phase (A_600_∼0.5) in LB medium at 32°C; 30 min prior to imaging, cultures were treated with vancomycin hydrochloride (1.5 µg/ml).

Cell structures were stained using membrane dye FM4-64 (Molecular Probes; 1 µg/ml) or of DAPI/ml (250 ng/ml), and 1.5 µl of cell suspension was spotted onto a glass slide for microscopy. Digital images were captured using a Quorum WaveFX Spinning Disc Confocal System. Cell length was measured using the Volocity program (Improvision Ltd.).

For drug interaction assays, drugs were tested over varying concentration ranges. The minimum inhibitory drug concentration was deemed to substantially reduce the growth rate by at least 20–50% relative to a no-drug control. Drug combinations were compared to single drugs using an isobologram on an arithmetic scale. For further details, see Protocol S11.

### LPS/OM and ampicillin assays

The *in vitro* LPS purification and binding assay was based on an approach reported previously [67]. Total soluble LPS was separated by SDS-PAGE and examined by silver staining (see Protocol S12). Defects in OM protein biogenesis were assayed as previously described [26].

To assess ampicillin sensitivity, overnight cultures were diluted in fresh LB and grown at 32°C to an OD_600_ of ∼0.6. The cells were then diluted 1∶3 in fresh LB medium with or without 5 µg of ampicillin/ml and growth continued at 32°C, with OD_600_ measured every 20 min.

### Electron microscopy

Cells were fixed with 2% glutaraldehyde in 0.1 M sodium cacodylate buffer overnight, post-fixed with 1% osmium tetroxide, dehydrated in a graded ethanol series followed by absolute propylene oxide, and embedded in Quetol-Spurr resin. Sections (90 nm thick) were cut on a RMC MT6000 ultramicrotome, stained with uranyl acetate and lead citrate, and viewed on a FEI CM100 TEM.

### Quantitative real-time PCR (qRT-PCR)

To measure mRNA levels, target RNA was isolated from stationary phase mutant cultures grown in rich or minimal medium using standard hot phenol extraction procedure, followed by DNase I treatment. cDNA was synthesized from ∼0.5 µg total RNA using iScript™ cDNA synthesis kit with SYBR green supermix (Bio-Rad) following the manufacture's protocol. Primer sequences used for qRT-PCR were as follows: forward primer of *cls* gene, 5′ GATTATATTTCGCGTTCACGTCTG-3′, reverse primer of *cls* gene, 5′-TCCGACTAACGGCAGAATGTAA-3′; forward primer of *ybaY* gene, 5′ATTACCGTGAATGACAAACTGGTA-3′, reverse primer of *ybaY* gene, 5′- CAGGTTGTTGTGTTGCTGAAATAG-3′; forward primer of *mtr* gene, 5′- TCTGCATCACACCTTCGCAGAGAT-3′, reverse primer of *mtr* gene, 5′-TTACGCCAAGGAACGAACTCGCTA -3′; forward primer of *ompW* gene, 5′-GCGGCTTTGGCAGTAACAACTCTT-3′, reverse primer of *ompW* gene, 5′-TGATGAACGGTTGCAATATCGCCG-3′; forward primer of *entB* gene, CGCGACTACTGCAAACAGCACAAT-3′ and reverse primer of *entB* gene, 5′-ATCAGGTTGTCGTCATCGAACGGT-3′. A primer set specific to glutathione S- transferase (*gst*) (forward primer of *gst* gene, 5′- CTTTGCCGTTAA CCCTAAGGG -3′; reverse primer of *gst* gene, 5′- GCTGCAATGTGCTCTAACCC -3′) was used as an internal control for normalization. mRNA quantification was performed on a Rotor-Gene RG-300 qRT-PCR system (Corbett Research) under standard reaction conditions. Relative mRNA expression level were quantified by comparing the cycle threshold (*C*_t_) values of each deletion mutant strain in rich medium or minimal medium to the *C*_t_ value of WT strain RNA, normalizing the sample value to *gst* expression. All reactions were performed independently from three biological replicates.

## Supporting Information

Figure S1 Conservation of envelope bioprocess components across 1078 bacteria grouped by taxa. Orthology relationships were determined through the InParanoid algorithm. Bacterial proteome datasets were obtained from the microbial genomes resource at the National Center for Biotechnology Information. Clustering of conservation profiles was performed using the open source software Cluster 3.0.
(TIF)

Figure S2 Analysis on the genetic interaction networks derived from two growth conditions. (A) Correlation of the normalized double mutant colony sizes between two replicates from each screen in rich or minimal medium. (B) Correlation of the normalized double mutant colony sizes produced by crossing 30 diverse F- ‘recipient’ strains with the corresponding set of 30 ‘donor’ mutants in either an Hfr C or an isogenic strain background. (C) Histogram of *E*-scores recorded on rich LB (RM) and on minimal medium (MM) growth conditions (32°C), with tails indicating aggravating and alleviating interactions. (D) Number of significant genetic interactions that occur between genes annotated to different pathways, as the |*E* - score| threshold is varied. The maximum number of enriched bioprocess pairs in the randomized data represents not more than 5% of the number of enriched bioprocesses in the actual dataset. (E, F) The distribution of genetic interaction network degree (panel E) and the betweenness (panel F) centrality measure, shown in a Log_2_ scale, for non-essential and essential genes in the filtered, high-confidence genetic interaction networks derived from rich (RM) and minimal (MM) medium growth conditions. P-values were computed using the Wilcoxon rank sum test (panel D), and the KS test (panel E).
(TIF)

Figure S3 Comparison of genetic interaction to the loss-of-fitness phenotype from chemogenomic study or to the mRNA expression levels derived from two growth conditions. (A) The percentage aggravating (panel I) or alleviating (panel II) genetic interactions from the double mutants involving essential or non-essential genes showing the loss-of -fitness phenotype from the study of Gross and colleagues [2]. (B) The network degree distribution of significant genetic interactions (|*E*-score|≥2) from rich and minimal medium culture conditions are shown for genes expressed at low (<7 normalized RMA units) and high (≥7 normalized RMA units) levels. The *p*-value is computed using Wilcoxon rank sum test.
(TIF)

Figure S4 Association of predicted envelope bioprocesses to alternate functional networks. Interactions derived from protein-protein interactions (A), gene co-expression (B), or genomic context (C) are shown for the 20 broadly representative functional bioprocesses.
(TIF)

Figure S5 Analysis on the correlation profiles of the multidrug resistance uptake systems. Pairwise correlation coefficients were computed for each mutant compared to the profiles generated from *mdtJ* or *mdtI* and from members of resistance-nodulation-cell division (RND), the major facilitator super family (MFS), multidrug and toxic compound extrusion (MATE) and ATP binding cassette (ABC) family linked to multidrug resistance.
(TIF)

Figure S6 Analysis on differential genetic interactions. (A) Enrichment of differential genetic interactions in rich versus minimal medium is shown for 20 broadly representative functional bioprocesses. (B) The scatterplot shows the number of positive and negative differential interactions associated with each gene targeted in this study. Genes with known and uncertain function that participate in opposing differential interactions is shown in the bottom panel.
(TIF)

Figure S7 Assignment of YhjD in LPS transport and YebA/YceG in septal PG splitting. (A) Chaining defects (arrows, panel I) caused by the indicated *yebA* and *yceG* double mutant and their respective single mutants. Strains stained with DAPI were visualized using a high content microscopy with differential interference contrast (DIC) and fluorescence optics. Gene rescue (suppression) is achieved by plasmid-based over-expression of *yebA* or *yceG* (panel II). Scale bar equals 2 µm. Cell length of indicated double and single mutants grown on rich medium is measured at 32°C (panel III). Error bars indicate the standard deviation of measurements from three independent experiments. (B) Full spectrum of *yhjD* genetic interaction and the indicated *yhjD*-*lpt* double mutants with a genetic interaction score. The gene pair (*yhjD*-*lptE*) showing a neutral interaction type is highlighted in bold text. (C) Growth of the indicated *yhjD* and LPS transport strains in liquid (LB) rich medium at 32°C over 24 h.
(TIF)

Protocol S1 Inclusion of hypomorphic essential alleles in the target selection to identify additional genetic interactions.
(PDF)

Protocol S2 Construction of *E. coli* cell-envelope genetic interaction map.
(PDF)

Protocol S3 Data processing and quality control.
(PDF)

Protocol S4 Generation of genetic interaction *E*-score.
(PDF)

Protocol S5 SPA-tag essential hypomorphs.
(PDF)

Protocol S6 Differences between our method and that of Ideker's method.
(PDF)

Protocol S7 Dataset for comparing network properties in both growth conditions.
(PDF)

Protocol S8 Construction of functional links between the bioprocesses using physical association, co-expression and genomic context.
(PDF)

Protocol S9 Immunoprecipitation.
(PDF)

Protocol S10 Phenotypic assays.
(PDF)

Protocol S11 Drug interaction assay.
(PDF)

Protocol S12 *In vitro* LPS binding assay.
(PDF)

Table S1 List of 821 target genes subject to eSGA screens.
(XLSX)

Table S2 Evolutionary conservation map of target genes belonging to twenty functional bioprocesses screened on rich or on minimal medium growth conditions based on the co-occurrence of putative orthologs across fully sequenced prokaryotic genomes.
(XLSX)

Table S3 List of gene pairs with associated significant genetic interaction scores in rich LB and in minimal medium growth conditions.
(XLSX)

Table S4 Genes from the rich medium screens with single or multiple annotated functions showing synthetic lethal interactions.
(XLSX)

Table S5 Comparison of genetic interactions from two different growth conditions (rich and minimal medium, this study) to literature curated cell envelope gene pairs from low-throughput experimental studies.
(XLSX)

Table S6 Correlation coefficients for the genetic interaction profiles of each gene across all other genes in rich LB and in minimal medium growth conditions.
(XLSX)

Table S7 Enrichment analysis on genetic interaction cross talk between various functional bioprocess combinations in rich medium.
(XLSX)

Table S8 Enrichment analysis on genetic interaction cross talk between various functional bioprocess combinations in minimal medium.
(XLSX)

Table S9 Differential genetic interactions significant in rich medium and in minimal medium genetic screens.
(XLSX)

Table S10 Known and predicted mRNA targets showing alleviating phenotypes with their cognate sRNA regulators.
(XLSX)

Table S11 Bacterial strains used in this study.
(XLSX)
