# Supplementary figures and images for: Genetic Interaction Maps in Escherichia coli Reveal Functional Crosstalk among Cell Envelope Biogenesis Pathways
Source: PLoS Genet. 2011 Nov 17;7(11):e1002377. doi: 10.1371/journal.pgen.1002377 (PMC3219608; doi:10.1371/journal.pgen.1002377)

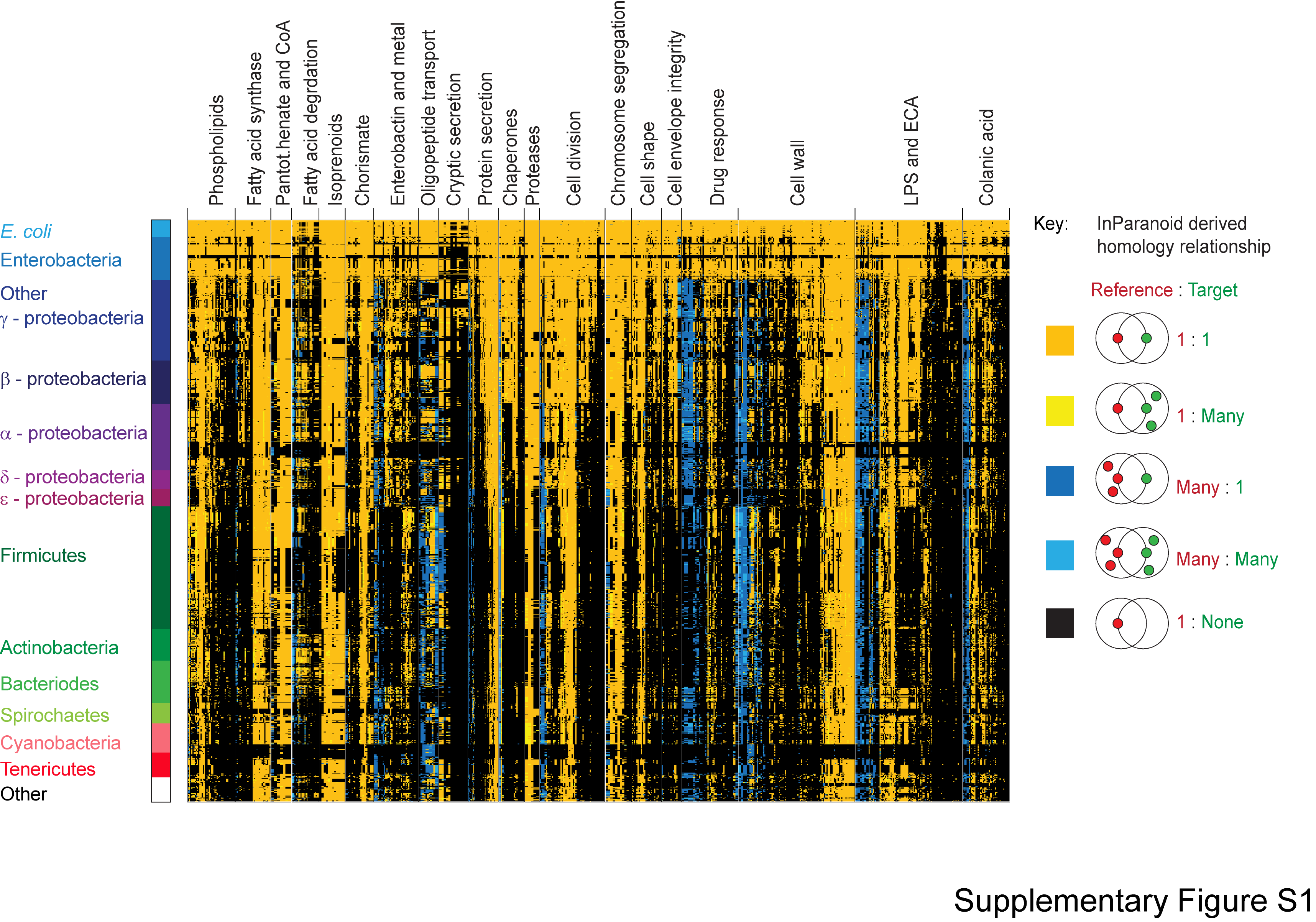

Supplement: Figure S1 — Conservation of envelope bioprocess components across 1078 bacteria grouped by taxa. Orthology relationships were determined through the InParanoid algorithm. Bacterial proteome datasets were obtained from the microbial genomes resource at the National Center for Biotechnology Information. Clustering of conservation profiles was performed using the open source software Cluster 3.0. (TIF) [file pgen.1002377.s001.tif]

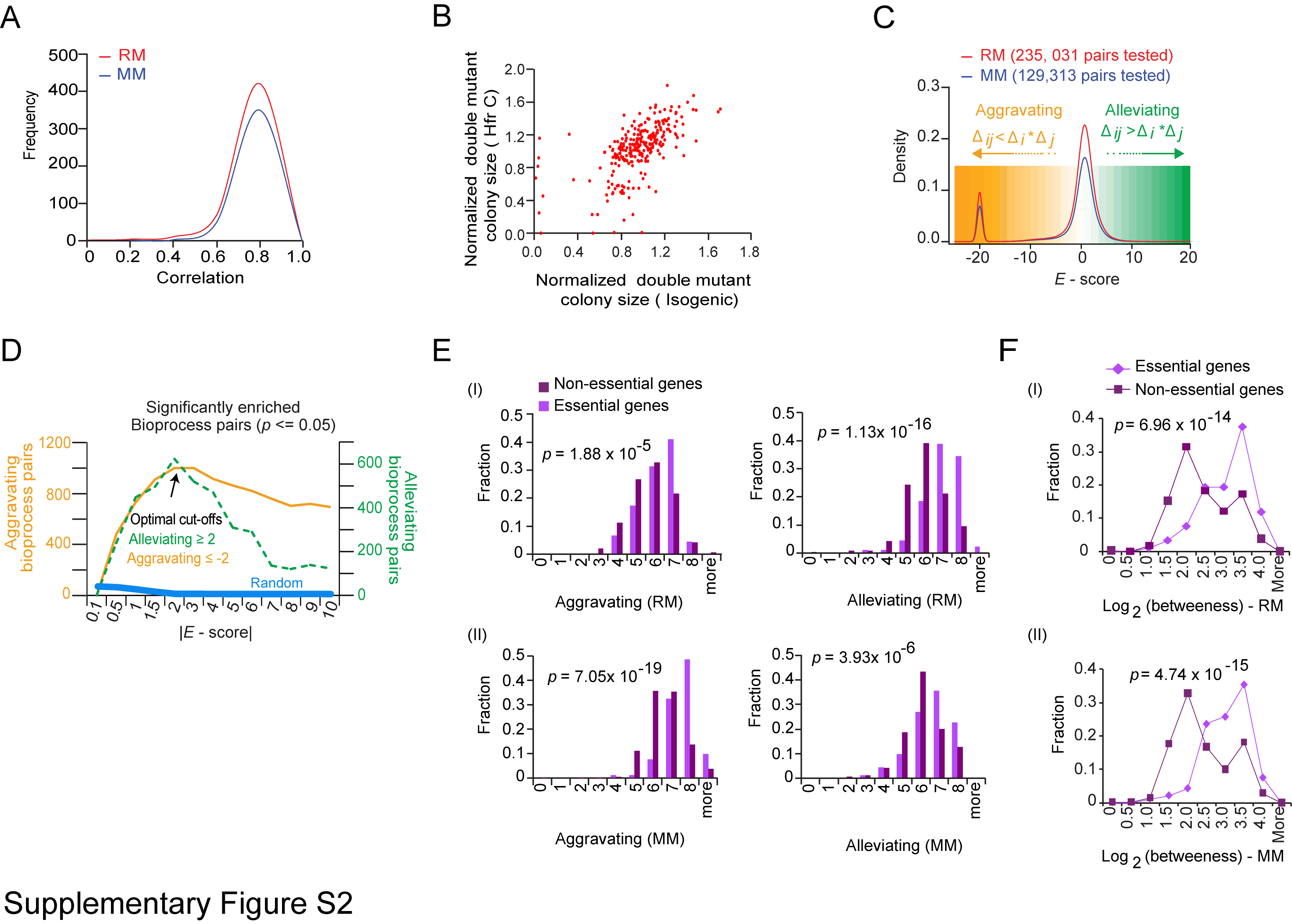

Supplement: Figure S2 — Analysis on the genetic interaction networks derived from two growth conditions. (A) Correlation of the normalized double mutant colony sizes between two replicates from each screen in rich or minimal medium. (B) Correlation of the normalized double mutant colony sizes produced by crossing 30 diverse F- ‘recipient’ strains with the corresponding set of 30 ‘donor’ mutants in either an Hfr C or an isogenic strain background. (C) Histogram of E-scores recorded on rich LB (RM) and on minimal medium (MM) growth conditions (32°C), with tails indicating aggravating and alleviating interactions. (D) Number of significant genetic interactions that occur between genes annotated to different pathways, as the |E - score| threshold is varied. The maximum number of enriched bioprocess pairs in the randomized data represents not more than 5% of the number of enriched bioprocesses in the actual dataset. (E, F) The distribution of genetic interaction network degree (panel E) and the betweenness (panel F) centrality measure, shown in a Log2 scale, for non-essential and essential genes in the filtered, high-confidence genetic interaction networks derived from rich (RM) and minimal (MM) medium growth conditions. P-values were computed using the Wilcoxon rank sum test (panel D), and the KS test (panel E). (TIF) [file pgen.1002377.s002.tif]

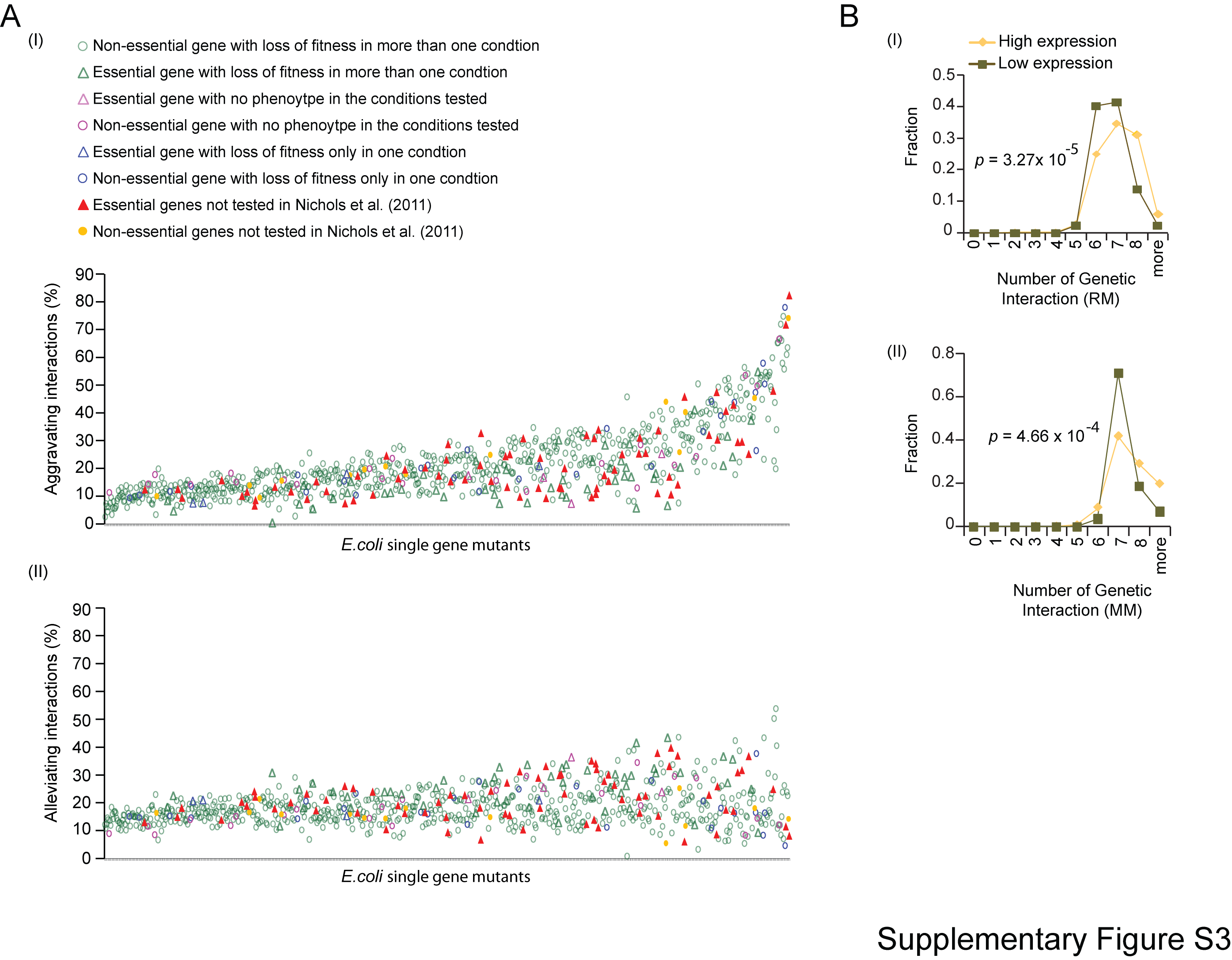

Supplement: Figure S3 — Comparison of genetic interaction to the loss-of-fitness phenotype from chemogenomic study or to the mRNA expression levels derived from two growth conditions. (A) The percentage aggravating (panel I) or alleviating (panel II) genetic interactions from the double mutants involving essential or non-essential genes showing the loss-of -fitness phenotype from the study of Gross and colleagues [2]. (B) The network degree distribution of significant genetic interactions (|E-score|≥2) from rich and minimal medium culture conditions are shown for genes expressed at low (<7 normalized RMA units) and high (≥7 normalized RMA units) levels. The p-value is computed using Wilcoxon rank sum test. (TIF) [file pgen.1002377.s003.tif]

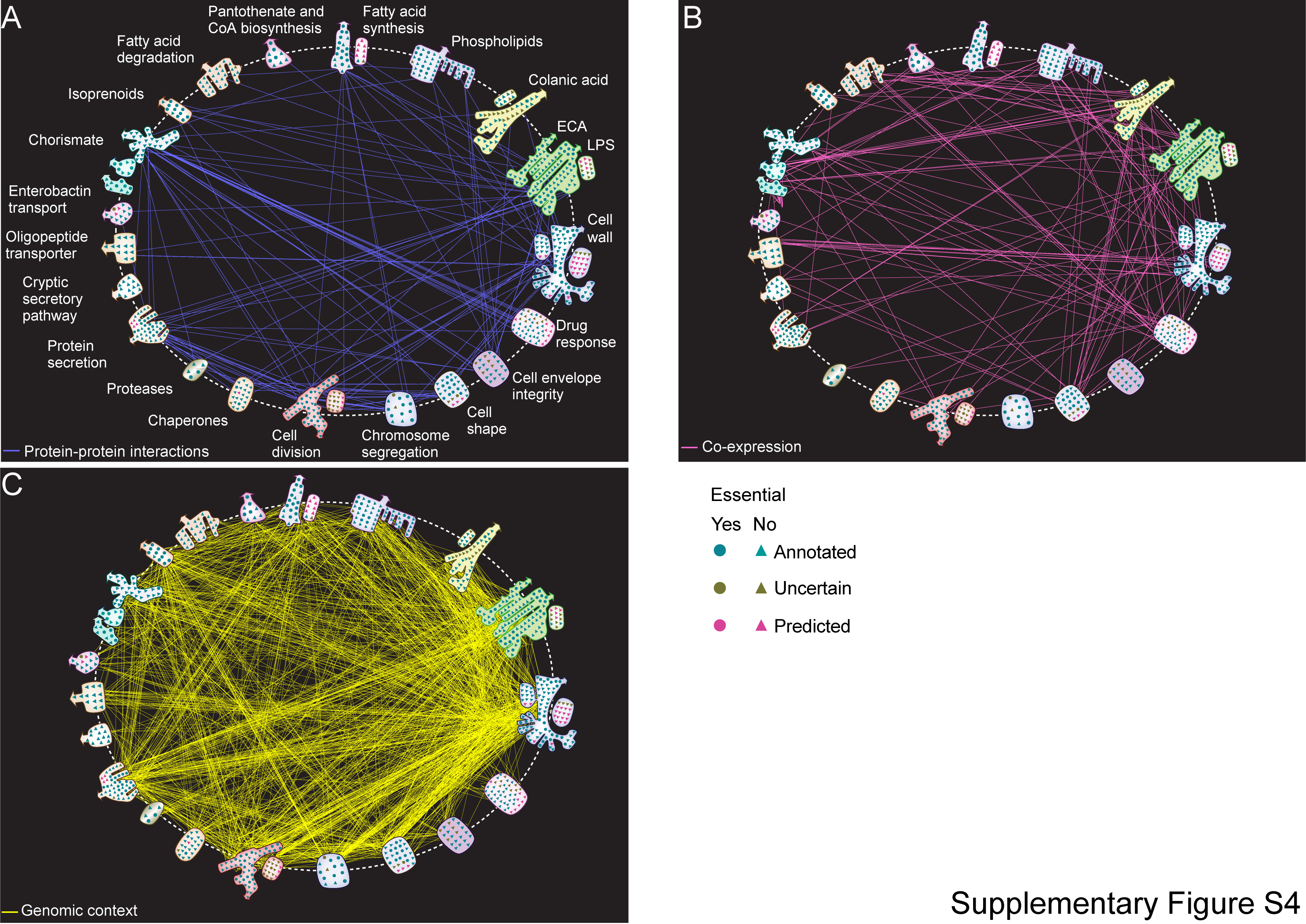

Supplement: Figure S4 — Association of predicted envelope bioprocesses to alternate functional networks. Interactions derived from protein-protein interactions (A), gene co-expression (B), or genomic context (C) are shown for the 20 broadly representative functional bioprocesses. (TIF) [file pgen.1002377.s004.tif]

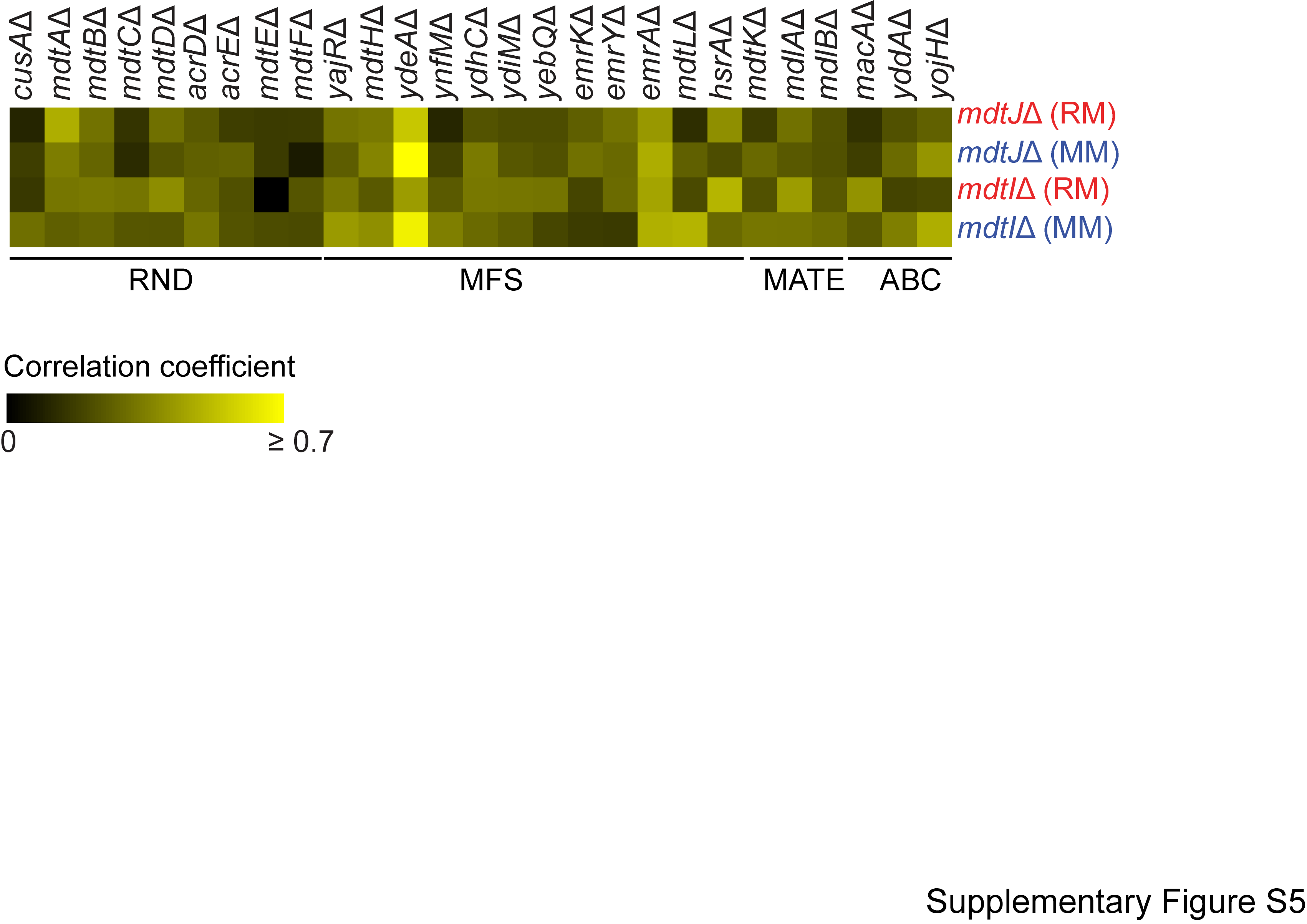

Supplement: Figure S5 — Analysis on the correlation profiles of the multidrug resistance uptake systems. Pairwise correlation coefficients were computed for each mutant compared to the profiles generated from mdtJ or mdtI and from members of resistance-nodulation-cell division (RND), the major facilitator super family (MFS), multidrug and toxic compound extrusion (MATE) and ATP binding cassette (ABC) family linked to multidrug resistance. (TIF) [file pgen.1002377.s005.tif]

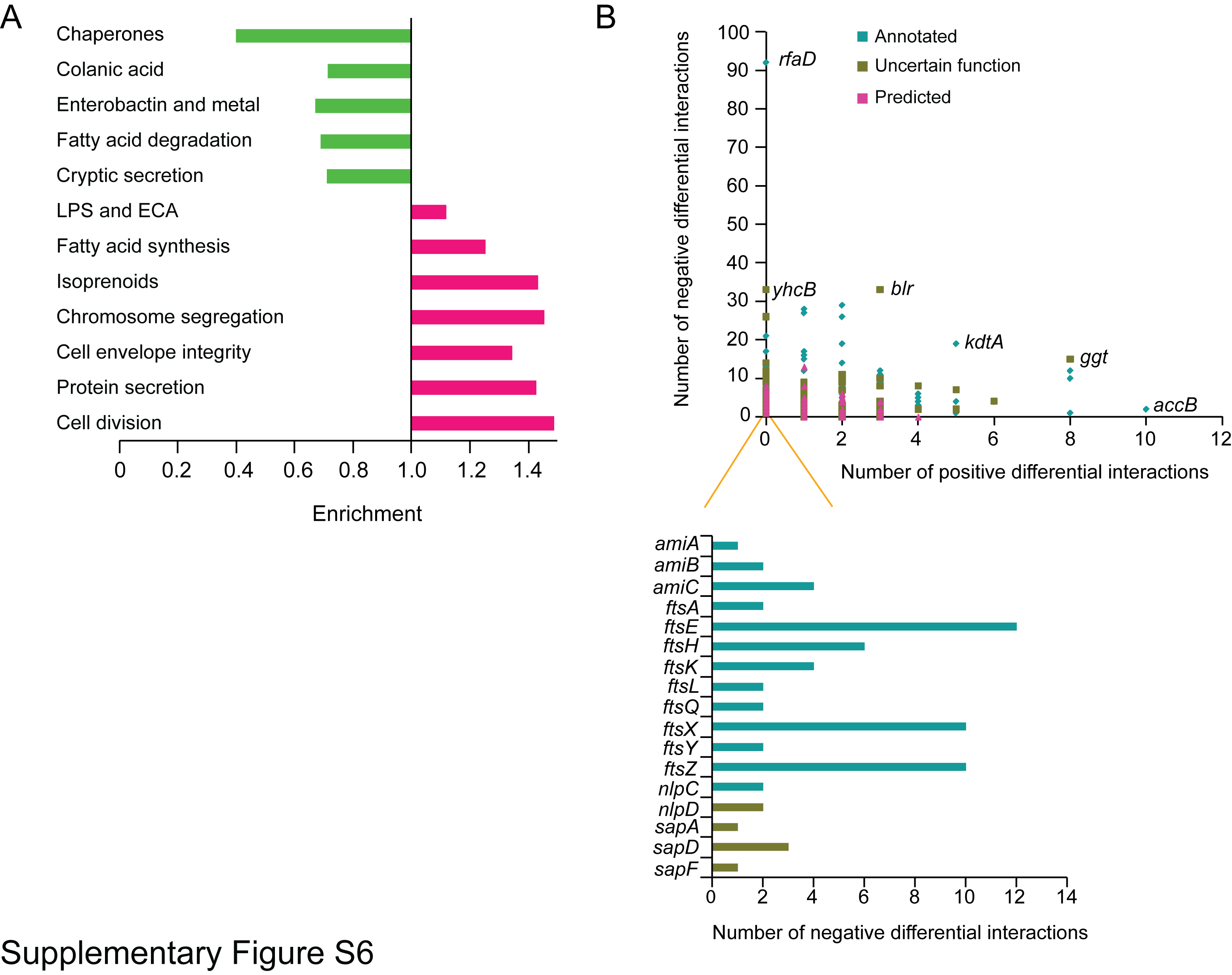

Supplement: Figure S6 — Analysis on differential genetic interactions. (A) Enrichment of differential genetic interactions in rich versus minimal medium is shown for 20 broadly representative functional bioprocesses. (B) The scatterplot shows the number of positive and negative differential interactions associated with each gene targeted in this study. Genes with known and uncertain function that participate in opposing differential interactions is shown in the bottom panel. (TIF) [file pgen.1002377.s006.tif]

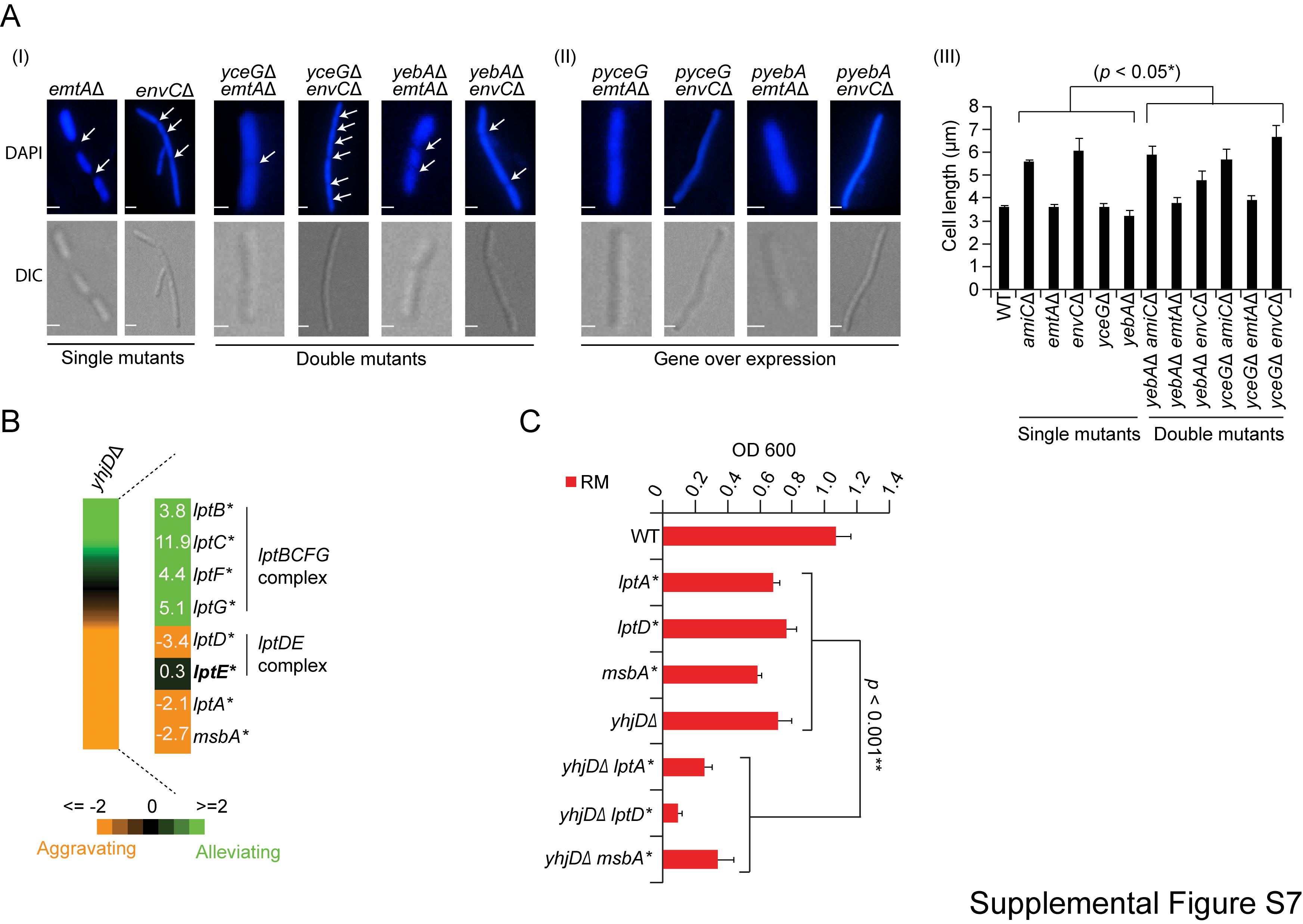

Supplement: Figure S7 — Assignment of YhjD in LPS transport and YebA/YceG in septal PG splitting. (A) Chaining defects (arrows, panel I) caused by the indicated yebA and yceG double mutant and their respective single mutants. Strains stained with DAPI were visualized using a high content microscopy with differential interference contrast (DIC) and fluorescence optics. Gene rescue (suppression) is achieved by plasmid-based over-expression of yebA or yceG (panel II). Scale bar equals 2 µm. Cell length of indicated double and single mutants grown on rich medium is measured at 32°C (panel III). Error bars indicate the standard deviation of measurements from three independent experiments. (B) Full spectrum of yhjD genetic interaction and the indicated yhjD-lpt double mutants with a genetic interaction score. The gene pair (yhjD-lptE) showing a neutral interaction type is highlighted in bold text. (C) Growth of the indicated yhjD and LPS transport strains in liquid (LB) rich medium at 32°C over 24 h. (TIF) [file pgen.1002377.s007.tif]
