# Supplementary material for: Genetic Interaction Maps in Escherichia coli Reveal Functional Crosstalk among Cell Envelope Biogenesis Pathways
Source: PLoS Genet. 2011 Nov 17;7(11):e1002377. doi: 10.1371/journal.pgen.1002377 (PMC3219608; doi:10.1371/journal.pgen.1002377)
Supplement: Protocol S1 — Inclusion of hypomorphic essential alleles in the target selection to identify additional genetic interactions. (PDF) [file pgen.1002377.s008.pdf]

Protocol S1. Inclusion of hypomorphic essential alleles in the target selection to identify additional genetic interactions

Since the F- recipient single gene knockout library (i.e. the 'Keio' collection) is comprised of only non-essential gene deletion mutants, we opted to use hypomorphic alleles (i.e. alleles with reduced gene function) to systematically assess the genetic interaction patterns of essential genes. This hypomorphic approach has also been performed in yeast using temperature-sensitive conditional alleles [1] and mRNA perturbation (DAmP) alleles [2]. In the current study, we screened 128 essential Hfr 'donor' or F- 'recipient' cell envelope strains that were potentially hypomorphic due to the presence of a C-terminal SPA (Sequential Peptide Affinity) tag extension engineered by homologous recombination into each gene. We reasoned that the alteration of the 3'-UTR *via* integration of the marker cassette could destabilize certain transcripts, or impede the proper function of the fusion protein. Although we were successful in SPA-tagging all of the 128 essential *E. coli* cell envelope proteins without any obvious pronounced growth defects, minor detrimental effects resulting from the SPA tag integration should become evident when other functionally linked components are compromised (by mutational inactivation in the same or another pathway). Indeed, a hypomorphic was independently reported for most (54/58) of these SPA-tag essential strains tested in a recent study by Carol Gross and colleagues [3] who evaluated culture condition-dependent slow growth phenotypes associated with *E. coli* strains [3].

References:

1. Li Z, Vizeacoumar FJ, Bahr S, Li J, Warringer J, et al. (2011) Systematic exploration of essential yeast gene function with temperature-sensitive mutants. Nat Biotechnol 29: 361-367.
2. Davierwala AP, Haynes J, Li Z, Brost RL, Robinson MD, et al. (2005) The synthetic genetic interaction spectrum of essential genes. Nat Genet 37: 1147-1152.

3. Nichols RJ, Sen S, Choo YJ, Beltrao P, Zietek M, et al. (2011) Phenotypic landscape of a bacterial cell. Cell 144: 143-156.
