# Supplementary material for: Genetic Interaction Maps in Escherichia coli Reveal Functional Crosstalk among Cell Envelope Biogenesis Pathways
Source: PLoS Genet. 2011 Nov 17;7(11):e1002377. doi: 10.1371/journal.pgen.1002377 (PMC3219608; doi:10.1371/journal.pgen.1002377)
Supplement: Protocol S2 — Construction of E. coli cell-envelope genetic interaction map. (PDF) [file pgen.1002377.s009.pdf]

Protocol S2. Construction of *E. coli* cell-envelope genetic interaction map

As in eSGA[1], each Hfr C cell-envelope donor, marked with Cm^R was grown in 384 colony format on LB medium, and then robotically pinned, in duplicate, onto Kan^R marked F^- recipients arrayed at 384-spot density. After ~24 hrs of conjugation at 32 ºC, the resulting double mutants are replica pinned at 384-colony density and subjected to double antibiotic (Cm and Kan) selection on the LB medium. The surviving double mutants on the rich LB medium were replica pinned onto the minimal medium to identify condition specific genetic interactions.

While it may be true that selecting the surviving double mutants from minimal medium and replica pinning them on-to rich LB medium might reveal new interactions, extensive previously unpublished work during the development of the eSGA method revealed the use of rich medium for the conjugation step resulted in more consistent growth of F- recipient gene deletion mutant strains in a reasonable screening time frame. Cells grown in minimal medium not only required much longer incubation times (approximately 2 weeks to complete an eSGA screen when pinned onto minimal medium versus <6 days using rich medium), but also displayed greater growth rate variability among the different recipient mutant strains and poorer reproducibility between replicate screens. Furthermore, this variability leads to inconsistent and less efficient conjugation when a constant number of donor cells were mated with varying numbers of recipient cells (depending on the viability of each F- recipient mutant in minimal medium). Hence, we opted for a more reliable screening strategy to minimize spurious variance while simplifying the study logistics.

Moreover, we observed that the long conjugation time partially averted the problem of poor transfer efficiency of markers distal to *OriT* [1]. For reproducibility purpose, each donor

strain was screened independently twice against the recipient array collection to test each combination of mutations in duplicate.

The standard growth, mating and selection of strains, unless otherwise specified, were done at 32°C. This temperature was chosen because both the non-essential deletion[1] and the hypomorphic-essential, Cm^R-marked, donor Hfr Cavalli strains, as well as the hypomorphic, Kan^R-marked, recipient strains in F^- DY330 [1] have a partial λ temperature-inducible prophage under the control of a *cI857* temperature-sensitive repressor, which is repressed at 32°C and de-repressed at 42°C[2]. Expression of λ genes facilitates homologous recombination during strain construction[2]. Leakage in repression, which may occur between repression and de-repression temperatures, may affect strain fitness. Therefore, this temperature was chosen to avoid introducing bias in growth fitness.

References:

1. Butland G, Babu M, Díaz-Mejía JJ, Bohdana F, Phanse S, et al. (2008) eSGA: E. coli synthetic genetic array analysis. Nat Methods 5: 789-795.
2. Yu D, Ellis HM, Lee EC, Jenkins NA, Copeland NG, et al. (2000) An efficient recombination system for chromosome engineering in Escherichia coli. Proc Natl Acad Sci U S A 97: 5978-5983.
