# Supplementary material for: Genetic Interaction Maps in Escherichia coli Reveal Functional Crosstalk among Cell Envelope Biogenesis Pathways
Source: PLoS Genet. 2011 Nov 17;7(11):e1002377. doi: 10.1371/journal.pgen.1002377 (PMC3219608; doi:10.1371/journal.pgen.1002377)
Supplement: Protocol S3 — Data processing and quality control. (PDF) [file pgen.1002377.s010.pdf]

Protocol S3. Data processing and quality control

Plate image processing and colony size quantization were adapted from an automated image processing system originally devised for yeast[1]. We minimized false positives (spurious interactions) and false negatives (missed interactions) by correcting for both batch and plate effects (e.g. asymmetries stemming from colony coordinates) and the intrinsic growth rates of single mutants. Briefly, the average fitness of a given recipient strain (measured across all donors) was calculated and normalized to the corresponding average fitness of the same allele in the donor strain (measured across all recipients). By comparing the phenotype obtained for a particular double mutant against all other strain combinations involving the same individual alleles, more accurate measures of both the directions and strengths of putative genetic interaction were obtained. Importantly, both the raw colony sizes and the normalized growth fitness of the digenic mutants generated between the donor mutant replicates from different days of pinning virtually gave identical results (data not shown), underlining the reliability of the screening procedure.

Data quality was improved by correcting the typically larger colony sizes observed at the outermost edges of a plate (i.e. due to more available medium) relative to the centre of the plate (i.e. where colonies compete for limiting nutrients). Additional potential sources of systematic error that arose from unusual noisiness, pinning defects, or otherwise unreliable experiments were eliminated. Experiments were considered unreliable if a substantial fraction of colonies were missing, or if the colony sizes among the duplicate experimental plates were not correlated. We also filtered cases where the growth fitness measurement of the double mutant generated from the recipient F^- single gene deletion mutants obtained from the Keio collection were defective for genetic duplications or unknown compensatory lesions[2]. We also removed donor

mutants exhibiting a mucoid phenotype (i.e. slimy colony), because the resulting strains gave inconsistent colony size measurements that did not reflect actual growth fitness.

Prior to performing the eSGA genetic screens, only viable non-essential and hypomorphic gene mutant strains in both the Hfr 'donor' and F- 'recipient' strains, respectively, were considered suitable for studying synthetic phenotypes. Single mutant strains that had either a severe growth fitness defect (i.e. noticeable slow growth) or mutations that gave rise to phenotypic changes affecting colony morphology (i.e. mucoidity) were not included in the screening process. In some of the strains screened, single mutant growth was relatively poor, leading to uniformly slow growth among most of corresponding double mutants. However, in these cases, the resulting *E*-score are not significant, as the deviation from the expected multiplicative phenotype would be relatively small and universal.

Despite having many hypomorphs according to Gross et al "Cell" study of our SPA-tagged strains [3], we also note that our SPA-tag essential gene alleles tended to show fewer aggravating interactions on average than non-essential genes in our screens (as indicated in Figure S3A). Moreover, whereas 51% of double mutants involving hypomorphic essential mutant gene pairs showed alleviating interactions, only 12% had aggravating relationships (i.e. showed less than expected combined fitness of the two mutations). That is, "sick plus sick equals dead" was relatively rare for essential genes, and was in fact was no more likely for essential-essential double mutant combinations.

While follow up experiments are necessary to account for the mechanistic basis behind the 12% of the aggravating hypomorphic pairs, these results nevertheless support the notion that that correlations among genetic profiles are informative regardless of occasional, less-specific interactions by certain mutants.

References:

1. Collins SR, Schuldiner M, Krogan NJ, Weissman JS (2006) A strategy for extracting and analyzing large-scale quantitative epistatic interaction data. Genome Biol 7: R63.
2. Yamamoto N, Nakahigashi K, Nakamichi T, Yoshino M, Takai Y, et al. (2009) Update on the Keio collection of Escherichia coli single-gene deletion mutants. Mol Syst Biol 5: 335.
3. Nichols RJ, Sen S, Choo YJ, Beltrao P, Zietek M, et al. (2011) Phenotypic landscape of a bacterial cell. Cell 144: 143-156.
