# Supplementary material for: Genetic Interaction Maps in Escherichia coli Reveal Functional Crosstalk among Cell Envelope Biogenesis Pathways
Source: PLoS Genet. 2011 Nov 17;7(11):e1002377. doi: 10.1371/journal.pgen.1002377 (PMC3219608; doi:10.1371/journal.pgen.1002377)
Supplement: Protocol S4 — Generation of genetic interaction E-score. (PDF) [file pgen.1002377.s011.pdf]

Protocol S4. Generation of genetic interaction *E*-score

Prior to the generation of *E*-score, the normalized colony sizes for each gene pair that were tested with two independent technical replicates were averaged and corrected by technical reproducibility to create one composite *E*-score. Thus, the final interaction data set subsequently used for functional analysis had two *E*-scores generated for each pair of double mutants that were tested reciprocally. In some cases, only one measurement of *E*-score was generated for some gene pairs that were not tested in both ways because we were unable to generate the double mutant fitness score (*E*-score). For example, in cases where the donor deletion strains expressing mucoidity failed to recombine with recipient deletion strains. Over all, in cases, where there was a substantial inconsistency, the gene pair with an *E*-score closer to zero or to the neutrality (i.e. double mutant fitness closer to the multiplicative fitness of the two respective single mutants) was chosen as a more conservative and reliable measure. Since query and recipient loci suffering from reduced recombination deficiency due to chromosomal proximity result in unreliable score fitness, we removed all recipient genes located within 30 kbp region of the query gene essentially as previously described[1].

Epistasis interactions were derived whenever a double mutant showed a significant deviation in fitness (W_{ij}) compared to the expected multiplicative effect of combining two single mutants[2] (W_i x W_j). Each pair of genes (*i*, *j*) was ranked with an *E*-score quantifying this deviation, as in equation 1.

$$\varepsilon_{ij} = W_{ij} - W_i \times W_j \,, \tag{1}$$

These fitness values derive from the mutant colony sizes, which first had to be corrected for systematic experimental variations. Raw colony sizes, S_{dr} of double mutants were measured

from processed digital images of the array plate containing donor *d* and recipient *r* – similar to eSGA[1]. We initially measured these sizes from a few plates over several time points between 16 to 48 hrs and found that they were linearly proportional to the length of the time course ($S_{dr} \propto \alpha_{dr} t$, where α_{dr} is the growth rate and t is is the growing time), and that these colony sizes showed high reproducibility [Pearson Correlation (PC) = 95%] during the linear phase of growth (data not shown). To account for the variation in colony sizes due to the location of the mutants on the array, nutrient competition, and experimental batch, we normalized the growth S_{dr} by a position-specific factor v_{dr}, to generate a final corrected colony size as in equation 2.

$$\tilde{S}_{ij} = \frac{S_{ij}}{v_{ij}} = C\alpha_{ij} t . \quad (2)$$

A constant factor for all colonies, *C*, accounts for experimental variations caused by growth competition and medium composition (e.g. rich and minimal medium).

The double mutant fitness is defined as the growth rate of the double mutant relative to wild type. Since genetic interactions are rare events, the growth rate of each donor were estimated as the mode of all double-mutant growth rates involving that donor. The wild type growth rate was then estimated as the mode of these single mutant donor growth rates:

$$W_{dr} = \frac{\alpha_{dr}}{\underset{d}{\text{mode}}\ \underset{r}{\text{mode}}\, \alpha_{dr}} = \frac{\tilde{S}_{dr}}{\underset{d}{\text{mode}}\ \underset{r}{\text{mode}}\, \tilde{S}_{dr}}$$

The single mutant fitness values in equation 1 were estimated using the colony sizes of single mutants pinned onto the same array plate. These sizes were normalized according to equation 2, where *d* was assumed constant and *r* varied for each mutant.

To account for the variances of each single and double mutant, we introduced the epistasis interaction *E*-score including the error estimation using the following formula:

$$\mathrm{E}_{dr} = \frac{\varepsilon_{dr}}{\sigma} = \frac{W_{dr} - W_d W_r}{\sqrt{\frac{\mathrm{var}_{dr}}{n_{dr}} + W_r^{\ 2} \frac{\mathrm{var}_d}{n_d} + W_d^{\ 2} \frac{\mathrm{var}_r}{n_r}}},$$

where, $\mathrm{var}_k = \sigma_k^2$ (k=d, r, dr) is the variance in fitness for the mutant of interest, σ_k is the standard deviation and n_k is the number of measurements of the fitness for the mutant of interest. Finally, the *E*-score for a pair of genes *i*, *j* was taken as the average of the E_{ij} and *Eji* except where E_{ij} and E_{ji} deviated significantly, in which case the minimum of the two scores was used.

The *P*-values for *E*-scores were evaluated by translating each *E*-score into a Z-score, with the median, Q_{50}, and scaled inter-quartile range, $(Q_{75} - Q_{25})/1.35$, replacing the mean and standard deviation - both to reduce the influence of outliers and to abrogate the influence of the heavy negative tail of the empirical *E*-score distribution. Each *p*-value was therefore calculated as:

$$P(x) = 2 * \varphi\left(\frac{x - Q_{50}}{(Q_{75} - Q_{25})/1.35}\right) \; for\, x < 0,$$

$$P(x) = 2 * \left[1 - \varphi\left(\frac{x - Q_{50}}{(Q_{75} - Q_{25})/1.35}\right)\right] \; otherwise.$$

Where φ is the cumulative distribution function of the standard normal distribution, and 1.35 is the approximate inter-quartile range of the standard normal distribution. The per-pathway enrichment for differential interactions was calculated as:

$$Enrichment = \frac{Fraction\ of\ within\ pathway\ GI\ that\ are\ differential}{Fraction\ of\ all\ other\ GI\ that\ are\ differential}$$

Significance was evaluated using the fisher exact test.

References:

1. Butland G, Babu M, Díaz-Mejía JJ, Bohdana F, Phanse S, et al. (2008) eSGA: E. coli synthetic genetic array analysis. Nat Methods 5: 789-795.
2. Segrè D, Deluna A, Church GM, Kishony R (2005) Modular epistasis in yeast metabolism. Nat Genet 37: 77-83.
