# Supplementary material for: Genetic Interaction Maps in Escherichia coli Reveal Functional Crosstalk among Cell Envelope Biogenesis Pathways
Source: PLoS Genet. 2011 Nov 17;7(11):e1002377. doi: 10.1371/journal.pgen.1002377 (PMC3219608; doi:10.1371/journal.pgen.1002377)
Supplement: Protocol S5 — SPA-tag essential hypomorphs. (PDF) [file pgen.1002377.s012.pdf]

Protocol S5. SPA-tag essential hypomorphs

Prior to performing the eSGA genetic screens, only viable non-essential and hypomorphic gene mutant strains in both the Hfr ‘donor’ and F- ‘recipient’ strains, respectively, were considered suitable for studying synthetic phenotypes. Single mutant strains that had either a severe growth fitness defect (i.e. noticeable slow growth) or mutations that gave rise to phenotypic changes affecting colony morphology (i.e. mucoidity) were not included in the screening process. In some of the strains screened, single mutant growth was relatively poor, leading to uniformly slow growth among most of corresponding double mutants. However, in these cases, the resulting *E*-score are not significant, as the deviation from the expected multiplicative phenotype would be relatively small and universal.

Despite having many hypomorphs according to Gross et al “Cell” study of our SPA-tagged strains [1], we also note that our SPA-tag essential gene alleles tended to show fewer aggravating interactions on average than non-essential genes in our screens (as indicated in Figure S3A). Moreover, whereas 51% of double mutants involving hypomorphic essential mutant gene pairs showed alleviating interactions, only 12% had aggravating relationships (i.e. showed less than expected combined fitness of the two mutations). That is, "sick plus sick equals dead" was relatively rare for essential genes, and was in fact was no more likely for essential-essential double mutant combinations.

While follow up experiments are necessary to account for the mechanistic basis behind the 12% of the aggravating hypomorphic pairs, these results nevertheless support the notion that that correlations among genetic profiles are informative regardless of occasional, less-specific interactions by certain mutants.

References:

1. Nichols RJ, Sen S, Choo YJ, Beltrao P, Zietek M, et al. (2011) Phenotypic landscape of a bacterial cell. Cell 144: 143-156.
