# Supplementary material for: Genetic Interaction Maps in Escherichia coli Reveal Functional Crosstalk among Cell Envelope Biogenesis Pathways
Source: PLoS Genet. 2011 Nov 17;7(11):e1002377. doi: 10.1371/journal.pgen.1002377 (PMC3219608; doi:10.1371/journal.pgen.1002377)
Supplement: Protocol S6 — Differences between our method and that of Ideker's method. (PDF) [file pgen.1002377.s013.pdf]

Protocol S6. Differences between our method and that of Ideker's method

Both our experimental and computational approach in many ways differs from the work of Ideker and colleagues [1]. In the experimental setup, Ideker and colleagues screened genetic interactions among double mutants in standard and in genotoxic stress-growth conditions separately, while we replica pinned only the surviving double mutants from rich media to minimal media. Thus, our dataset does not contain any information about strains that are lethal in rich media, but may be viable in minimal media.

In the computational analysis, our approach is simpler than Ideker's work – in that we only consider genetic interactions that change sign (i.e. from aggravating to alleviating, or *vice versa*) between the two conditions. Ideker work examined these interactions, and in addition interactions that did not change sign. For example, an interaction that was strongly alleviating in standard growth media but only very weakly alleviating or neutral in genotoxic stress condition may have been considered as a significant "differential" interaction.

Unlike in Ideker's method [1], we analyzed the rich and minimal media networks separately, to find commonalities as well as differences between the two condition-specific networks. Thus, while Ideker's method primarily highlights differential interactions related to DNA repair, our paper has a broader scope aimed at characterizing the functional relationships among components of the bacterial cell envelope. As such, we explore multiple facets of the genetic interaction network – including differential and non-differential interactions, genetic interaction profile correlation, network topology, experimental confirmation of epistatic relationships among multiple cell envelope subsystems, and pathway enrichment analysis.

References:

1. Bandyopadhyay S, Mehta M, Kuo D, Sung MK, Chuang R, et al. (2010) Rewiring of genetic networks in response to DNA damage. Science 330: 1385-1389.
