# Supplementary material for: Genetic Interaction Maps in Escherichia coli Reveal Functional Crosstalk among Cell Envelope Biogenesis Pathways
Source: PLoS Genet. 2011 Nov 17;7(11):e1002377. doi: 10.1371/journal.pgen.1002377 (PMC3219608; doi:10.1371/journal.pgen.1002377)
Supplement: Protocol S7 — Dataset for comparing network properties in both growth conditions. (PDF) [file pgen.1002377.s014.pdf]

Protocol S7. Dataset for comparing network properties in both growth conditions

Prior to filtering the networks, we deleted any pairs that were not tested in both networks (Table S3). New rich and minimal medium networks were then defined by filtering these data at |*E*-score| ≥ 2. These new, restricted networks were defined to contrast the properties of the rich and minimal medium networks for Figure2A; Figure S2E and S2F.

(a) Genetic interaction network properties and gene expression. Network betweenness (Figure S2F) was calculated using version 1.1 of the Centiscape[1] plug-in for Cytoscape version 2.6.3[2] on unweighted versions of the rich and minimal medium genetic interaction networks. These unweighted networks were derived by inferring an edge between two genes if the corresponding pair had an *E*-score ≥ 2, or an *E*-score ≤ -2. The significance of alleviating/aggravating ratio differences between essential genes and non-essential gene pairs in rich and in minimal medium (Figure 2A) was evaluated using the one-sided Fisher's exact test. Expression data for all the genes from wild type *E. coli* K-12 strain expressed in rich and in minimal medium was obtained from M3D database[3]. The expression signal for each gene is denoted in terms of normalized RMA (Robus Multi Array) values or units. Genes expressed at less than 7 units were assigned the category "Low Expression", and those expressed at 7 units or more were assigned the category "High Expression" (Figure S3B). Significance of rich and minimal medium genetic interactions versus high or low expression histograms, and the genetic interaction degree distribution for essential or non essential genes was evaluated using the one-sided Wilcoxon rank-sum test (Figure S2E and S3B). The *p*-values for the betweenness histograms were evaluated using the two-sample Kolmogorov-Smirnov (KS) test (Figure S2F).

(b) Comparison of genetic interaction profiles to high-quality *E. coli* PPI network, operons and co-expression. The histograms of uncentered Pearson correlation coefficients calculated for pairs of genetic interaction profiles were determined both for the 256 gene pairs annotated to belong to a common operon (Figure 2D) and for the 607 gene pairs coding for interacting proteins in the reference *E. coli* PPI Set (Figure 2F). These histograms were contrasted with pairs of genes randomly selected from among the genes tested in both rich and minimal medium. No score filtering was applied, and the correlation was computed between pairs of genetic interaction profiles – each derived from the concatenation of the genetic interaction profiles in rich and minimal medium. Scores that were undetermined were omitted during the calculation of pair-wise correlation. The random distribution was estimated by averaging 100 histograms, each computed from 256 (Figure 2D) or 607 (Figure 2F) randomly drawn pairs of genes. P-values were evaluated using the one-sided 2-sample KS test to compare the distribution of within operon or PPI pairs to that of all other pairs. For co-expression, the probability densities were estimated using Matlab "ksdensity" function', using default parameters.

References:

1. Scardoni G, Petterlini M, Laudanna C (2009) Analyzing biological network parameters with CentiScaPe. Bioinformatics 25: 2857-2859.
2. Shannon P, Markiel A, Ozier O, Baliga NS, Wang JT, et al. (2003) Cytoscape: a software environment for integrated models of biomolecular interaction networks. Genome Res. pp. 2498-2504.
3. Faith JJ, Driscoll ME, Fusaro VA, Cosgrove EJ, Hayete B, et al. (2008) Many Microbe Microarrays Database: uniformly normalized Affymetrix compendia with structured experimental metadata. Nucleic Acids Res 36: D866-870.
