# Supplementary material for: Genetic Interaction Maps in Escherichia coli Reveal Functional Crosstalk among Cell Envelope Biogenesis Pathways
Source: PLoS Genet. 2011 Nov 17;7(11):e1002377. doi: 10.1371/journal.pgen.1002377 (PMC3219608; doi:10.1371/journal.pgen.1002377)
Supplement: Protocol S8 — Construction of functional links between the bioprocesses using physical association, co-expression and genomic context. (PDF) [file pgen.1002377.s015.pdf]

Protocol S8. Construction of functional links between the bioprocesses using physical association, co-expression and genomic context

The PPI network was constructed by compiling a union of all the manually curated PPI data from the following databases: BIND, IntAct, MPI_LIT, eNET, and DIP. In the case of the co-expression network, ~350 representative expression microarrays were taken from the M3D database and filtered for redundancy. Arrays (conditions) with PC (Pearson correlation) ≥ 0.95 were considered as redundant and were filtered, resulting in ~115 non-redundant arrays. These arrays were then used to compute a PC for all versus all pairs of genes. Only gene pairs showing a correlation with a Z-score ≥ 2.58 ($p \leq 0.05$) from the mean of all gene pairs were considered as co-expressed. Using these co-expressed gene pairs, a network was constructed. In addition, we constructed a network of functional associations for *E. coli* cell envelope proteins by examining the natural chromosomal clustering of bacterial genes using GC methods, namely: (1) gene fusions; (2) similarity between phylogenetic profiles; (3) evolutionary conservation of gene order; and (4) intergenic distances. The network was created by integrating the data from all the four GC methods using Bayesian inference[1].

References:

1. Hu P, Janga SC, Babu M, Diaz-Mejia JJ, Butland G (2009) Global functional atlas of Escherichia coli encompassing previously uncharacterized proteins. PLoS Biol 7: e1000096.
