# Supplementary material for: Genetic Interaction Maps in Escherichia coli Reveal Functional Crosstalk among Cell Envelope Biogenesis Pathways
Source: PLoS Genet. 2011 Nov 17;7(11):e1002377. doi: 10.1371/journal.pgen.1002377 (PMC3219608; doi:10.1371/journal.pgen.1002377)
Supplement: Protocol S9 — Immunoprecipitation. (PDF) [file pgen.1002377.s016.pdf]

Protocol S9. Immunoprecipitation

The immunoprecipitation experiment was performed with an endogenously non-overproduced Kan marked SPA-tagged strain expressed in DY330 and with a Cm marked over-expressed His_6 tagged protein. One µl of the over expression plasmid (pCA24N) containing the full length His-tagged protein was transformed into the SPA-tagged strain. The transformants were selected on kanamycin (50 µg/ml) and chloramphenicol (34 µg/ml). The overnight *E. coli* DY330 strain harboring a SPA-tagged strain and a pCA24N plasmid containing the full length his-tag protein were grown at 25 ^{o}C in 50 ml terrific liquid broth containing kanamycin and chloramphenicol until it reaches OD_{600} ~ 0.6. The cells were centrifuged at 4,000 *xg* for 20 min and the pellets were resuspended in 50 ml of terrific liquid broth with appropriate antibiotics, followed by IPTG induction. The cells were induced for 3 h at 25 ^{o}C after the addition of 0.5 mM IPTG and harvested by centrifugation once again at 4,000 *xg*. Cells were then lysed *via* sonication essentially as previously described[1]. The immunoprecipitation was subsequently performed by incubating the sonicated cell lysates of DY330 strain expressing the SPA-tag strains in the presence ITPG as well as with 1% triton detergent for 3 hrs at 4°C with anti-Flag M2 agarose beads.

The immunoprecipitated proteins were finally separated on 10% SDS/PAGE and transferred onto nitrocellulose membranes as previously described[1]. The membranes were probed with polyclonal (LptD, OmpA, LamB, DegP) or monoclonal (MBP, LPS, His epitopes) antisera, and visualized by chemiluminescence (Pierce).

References:

1. Babu M, Butland G, Pogoutse O, Li J, Greenblatt JF, et al. (2009) Sequential peptide affinity purification system for the systematic isolation and identification of protein complexes from Escherichia coli. Methods Mol Biol 564: 373-400.
