# Supplementary material for: Genetic Interaction Maps in Escherichia coli Reveal Functional Crosstalk among Cell Envelope Biogenesis Pathways
Source: PLoS Genet. 2011 Nov 17;7(11):e1002377. doi: 10.1371/journal.pgen.1002377 (PMC3219608; doi:10.1371/journal.pgen.1002377)
Supplement: Protocol S10 — Phenotypic assays. (PDF) [file pgen.1002377.s017.pdf]

Protocol S10. Phenotypic assays

For drug assays, the exponentially growing deletion strains in LB medium were serially diluted and pinned onto LB plates in the absence or presence of the indicated concentration of the drugs such as polymyxin B (2 µg/ml) and amoxicillin (32 µM) or phosphomycin (16 µM) targeting LPS and cell wall processes, respectively. Sensitivity to the agent was assessed after 36 to 48 hrs of incubation at 32 °C. Ability of the gene deletion mutant strains defective for OM biogenesis was assayed using 6 mm filter paper disks impregnated with 5 µg of rifampin, and 5 µg of vancomycin by disk diffusion assay, essentially as previously described [1].

For gene rescue or complementation assay, the gene of interest were mini-prepped from the *E. coli* ASKA over expression plasmid library*[2]*. Plasmid pCA24N is a high-copy-number plasmid with a chloramphenicol resistance cassette for selection in *E. coli* and the isopropyl-β-d-thiogalactopyranoside (IPTG)-inducible promoter $P_{T5\text{-}lac}$*[2]*. The over expression plasmid was transformed into the deletion strain using standard molecular biology protocols.

References:

1. Ruiz N, Falcone B, Kahne D, Silhavy TJ (2005) Chemical conditionality: a genetic strategy to probe organelle assembly. Cell 121: 307-317.
2. Kitagawa M, Ara T, Arifuzzaman M, Ioka-Nakamichi T, Inamoto E, et al. (2005) Complete set of ORF clones of Escherichia coli ASKA library (a complete set of E. coli K-12 ORF archive): unique resources for biological research. DNA Res 12: 291-299.
