# Supplementary material for: Genetic Interaction Maps in Escherichia coli Reveal Functional Crosstalk among Cell Envelope Biogenesis Pathways
Source: PLoS Genet. 2011 Nov 17;7(11):e1002377. doi: 10.1371/journal.pgen.1002377 (PMC3219608; doi:10.1371/journal.pgen.1002377)
Supplement: Protocol S11 — Drug interaction assay. (PDF) [file pgen.1002377.s018.pdf]

Protocol S11. Drug interaction assay

For each drug, different ranges of concentrations were tested, where the minimum inhibitory concentration (MIC) of a drug was chosen when the bacterial growth had substantially reduced growth rate (20-50% reduction) relative to the no-drug control. To measure the combined effect of two drugs versus their individual effects, the wells were filled with 100 µl of total LB medium in the absence of drugs for control, and 80 µl of LB medium was inoculated with 10 µl of the overnight culture of the mutants or wild type cells with one of the following options: (a) 10 µl of 10 $_X$ stock of the drug X, to measure the growth rate of X singly; (b) the same for drug Y; (c) 10 µl of 10 $_X$ stock of drug X and 10 µl of 10 $_X$ stock of drug Y, to measure the two-drug growth rates. Comparison is made between the drugs in combination to those of the single drugs using an isobologram on an arithmetic scale where the graph is represented with the ratio of MIC of the drug X in combination to the MIC of the drug X when used alone in the x-axis and the ratio of the MIC of the drug Y in combination to the MIC of drug Y when used alone in the Y-axis. A straight line connecting the ratio 1.0 indicates the line of additivity. MIC of combination if it is located considerably below this line indicates synergy, above the line indicates antagonism and near the line indicates additive effect.
