# Supplementary material for: Genetic Interaction Maps in Escherichia coli Reveal Functional Crosstalk among Cell Envelope Biogenesis Pathways
Source: PLoS Genet. 2011 Nov 17;7(11):e1002377. doi: 10.1371/journal.pgen.1002377 (PMC3219608; doi:10.1371/journal.pgen.1002377)
Supplement: Protocol S12 — In vitro LPS binding assay. (PDF) [file pgen.1002377.s019.pdf]

Protocol S12. *In vitro* LPS binding assay

Prior to the *in vitro* LPS binding assay, the full-length YhjD, LptA or LolE was cloned into a pET41b plasmid harboring a kanamycin selectable marker with a C-terminal hexa-histidine (His_6) -tag under the control of a T7 promoter. The plasmid containing the full-length YhjD, LptA or LolE was transformed into BL21 (DE3) *E. coli* cells. The positive BL21 (DE3) *E. coli* cells harboring the YhjD, LptA or LolE pET41b plasmid were grown at 32 °C until OD_{600}~0.6. The cells were then placed for induction at 25 °C for 30 min with 0.5 mM IPTG. Induced cells were grown for additional 3 hrs at 25 °C and harvested by centrifugation at 4,000 *xg*. Cells were lysed *via* sonication in the lysis buffer containing 1% $C_{12}E_8$, 25 mM Tris (pH 8.0), 400 mM NaCl and protease inhibitors. The insoluble aggregates were subsequently removed by ultracentrifugation at 120,000 *xg*. Lysate was bound to 2mL of Qiagen Ni-NTA resin and washed twice with buffer A [0.1% $C_{12}E_8$, 25 mM Tris (pH 8.0), 400 mM NaCl and 30 mM imidazole]. The bound protein is eluted with the buffer B [25 mM Tris (pH 8.0), 400 mM NaCl] containing 400mM imidazole.

The *in vitro* LPS binding assay was based on the approach described previously[1] with slight modifications. The LPS from *E. coli* K-12 W3110 was extracted from the cell pellet using the phenol-chloroform-light petroleum (PCP) method[2]. Briefly, the *in vitro* binding assay was performed in 500 µl reaction volume in buffer B (25 mM Tris, pH 8.0, and 400 mM NaCl) containing 0.5 mg of purified YhjD, LolE or LptA- His_6 tag and 0.5 mg of the purified LPS. The mixture was incubated at 4 ºC for 2 hrs on a rotary shaker and passed through a column containing Ni-NTA resin (250 µl, washed in 1ml of the buffer B) and incubated again for additional 2 hrs. The resin was washed twice with 1.5 ml of the buffer B and then eluted with

500 µl of the buffer B containing 400 mM imidazole. The samples were separated by SDS-PAGE and examined by silver staining.

References:

1. Tran AX, Trent MS, Whitfield C (2008) The LptA protein of Escherichia coli is a periplasmic lipid A-binding protein involved in the lipopolysaccharide export pathway. J Biol Chem 283: 20342-20349.
2. Galanos C, Luderitz O, Westphal O (1969) A new method for the extraction of R lipopolysaccharides. Eur J Biochem 9: 245-249.
